# Taxonomic Studies on 10 Species Within the Genus *Impatiens* Based on the Complete Chloroplast Genomes and Morphological Researches, Along With the Report of Newly Discovered Species

**DOI:** 10.1002/ece3.73279

**Published:** 2026-03-18

**Authors:** Qinqin Yong, Xiao Wang, Meijun Li, Zhi Li, Xinxiang Bai, Sheng Liang, Jinling Zhang

**Affiliations:** ^1^ College of Forestry Guizhou University Guiyang China; ^2^ Guizhou Xishui National Nature Reserve Management Bureau Xishui China; ^3^ School of Tropical Agriculture and Forestry Hainan University Haikou China

**Keywords:** chloroplast genome, *Impatiens*, new species, phylogeny, synonym, *ycf1*

## Abstract

Southwestern China harbors high *Impatiens* diversity and numerous endemics, but its diversity and insufficient research have hindered clarification of their taxonomic and phylogenetic relationships. In this study, two distinct populations characterized by broadly funnelform lateral sepals were discovered in Guizhou. Given the complicated taxonomic relationships among their closely related species, further research is necessary to determine their exact taxonomic status. Investigations based on geographic proximity and morphological affinities tentatively linked the new populations to *Impatiens oxyanthera*, *I. piufanensis*, and its variety *I. forrestii*, as well as five other species. A taxonomic study of the aforementioned species was conducted using morphological comparison and genome sequencing (total of 10 chloroplast genomes). Phylogenetic analyses were performed based on the complete chloroplast genome sequences of 32 species across the Balsaminaceae, aiming to provide a basis for taxonomic decisions. The two new populations had 151,939 and 151,789 bp chloroplast genomes with a typical quadripartite structure, containing 113 genes. The *ycf1* was identified as a potential molecular marker. Phylogenetic analysis resolved three strongly supported clades (BS = 100). These 10 chloroplast genomes exhibit high pairwise homology. The two newly discovered populations with different flower color showed 99.7% identity. Combined with morphological verification, these two populations were confirmed as conspecific, and this taxon was formally named *Impatiens xishuiensis*. Taxonomic revisions include recognizing *I. piufanensis* and *I. forrestii* as synonyms of *I. oxyanthera*, *I. tienchuanensis* and *I. fanjingshanica* as synonyms of *I. commelinoides*. *I. sunii* is probably an infraspecific entity. Although morphologically similar to *I. oxyanthera*, *I. xishuiensis* is molecularly closer to *I. commelinoides*. Taxonomic revisions are corroborated by both genomic and morphological data. In *Impatiens*, floral color is likely of relatively limited value for inferring interspecific phylogenetic affinities. This study highlights the value of chloroplast genomes in resolving phylogenetic and taxonomic complexities in *Impatiens*.

## Graphical abstract

This study uses comparative chloroplast genomics and morphological analyses to clarify the phylogenetic positions of 10 morphologically similar *Impatiens* species in southwestern China, providing key taxonomic insights—including describing a new taxon (*Impatiens xishuiensis*) and proposing revisions—and advancing our understanding of karst‐adapted Impatiens speciation.

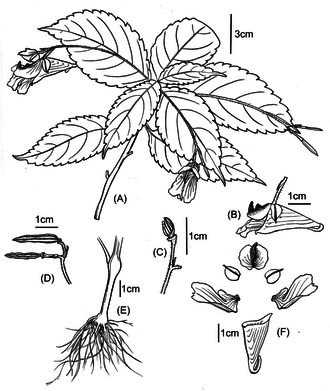

## 1 Introduction

The genus *Impatiens* L., established by the renowned Swedish botanist Linnaeus (1753), currently forms the family Balsaminaceae, together with the genus *Hydrocera* Bl. Globally, approximately 1200 species within this family have been documented, primarily distributed across the tropical and subtropical regions of the Old World (Grey‐Wilson 1980). In China alone, over 350 *Impatiens* species are known. Geographically, their distribution spans from tropical and subtropical regions to areas with higher latitudes and altitudes. Notably, the southern Xizang, the extensive Hengduan Mountains, the karst regions of Yunnan, Guizhou, and Guangxi, the Qinling‐Bashan Mountains, and the southeastern hilly areas are recognized as biodiversity hotspots for *Impatiens* in the country (Chen et al. 2023). In the late 19th century, the spread of Catholicism in southwestern China prompted a wave of Western botanists to explore southern China to collect plant specimens. This effort continued for half a century, with prominent researchers such as British botanist J. D. Hooker, Austrian botanist H. Handel‐Mazzetti, and French botanists A. R. Franchet and H. Léveillé contributing significantly to the study and publication of these specimens. However, due to the limitations of the era, early species descriptions were often based on single specimens or parts thereof. The lack of comprehensive population‐level field studies left the extent and patterns of morphological variation poorly understood, leading to blurred species boundaries. Additionally, factors such as the separation of collectors and researchers, limited specimen information, and changes in geographical nomenclature have further complicated taxonomic interpretations of early‐documented *Impatiens* species. These issues have posed significant challenges to subsequent research efforts (Huang et al. 2003; Chen 1978; Chen 1999). In recent years, increased field exploration has led to the discovery and documentation of numerous new taxonomic groups in southern China (Chen et al. 2023; Zeng et al. 2015; Lu et al. 2020; Peng et al. 2021; Song et al. 2021; Ren et al. 2022; Yuan et al. 2022; Huang, He, et al. 2023; Huang, Yuan, et al. 2023; Hu et al. 2024). The continual emergence of these new groups has highlighted the need for taxonomic revision of *Impatiens* species.

*Impatiens* species are renowned for their rich morphological diversity and high degree of convergent evolution. Their delicate floral parts often appear incomplete or folded in specimens, making them difficult to untangle and restore (Ruchisansakun et al. 2015; Rahelivololona et al. 2018). Consequently, the information retrieved from these specimens is often limited. Researchers worldwide have undertaken taxonomic treatments of *Impatiens* species, including synonyms consolidation and the establishment of new combinations. These efforts have primarily relied on macroscopic morphological traits, with limited attention given to molecular‐level analyses (e.g., Huang, He, et al. 2023; Huang, Yuan, et al. 2023; Singh et al. 2021). Due to the complex inter‐ and intra‐specific trait variations in *Impatiens* species, which are highly responsive to habitat changes, some taxonomic groups may have been over‐differentiated. As a result, issues such as synonyms and homonyms remain prevalent. The advent of molecular biology has seen the widespread application of chloroplast genomes in taxonomic research, resolving numerous taxonomic challenges at the family, genus, and closely related species levels (Yu et al. 2016; Nock et al. 2011; Bock 2007; Shaw et al. 2014; Yang et al. 2018; Schneider et al. 2021). Compared to nuclear and mitochondrial genomes, chloroplast genomes offer the advantage of being easier to sequence and assemble, yielding more abundant data resources. The large amount of nucleotide and amino‐acid sequence information within chloroplast genomes is invaluable for accurately discerning the genetic relationships among species. Traditional molecular systematics typically involves intensive sampling of a few molecular markers for systematic studies. In contrast, chloroplast genomes can complement and validate these studies, especially when the resolution is low or when molecular phylogenetic trees based on different markers show conflicting results (Zhang and Li 2011).

In 2016, Yu et al. constructed a taxonomic framework for the Balsaminaceae family using ITS and the *atpB‐rbcL* fragment of cpDNA. Building on this foundation, Qin et al. (2023) utilized chloroplast genomes to reconstruct the systematic framework of *I*. sect. *Impatiens*, offering more precise insights into phylogenetic relationships. Although some scholars have described and compared the chloroplast genomes of certain *Impatiens* species (Luo, Huang, et al. 2021; Luo, Yang, et al. 2021; Luo et al. 2022; Qiu et al. 2023), given the large number of *Impatiens* species, the sample size remains relatively small. Most studies focus on different sections within or between genera, leaving the relationships between closely related species largely unexplored.

The Yunnan‐Guizhou Plateau, which harbors the highest species richness of *Impatiens* in China, is an ancient floral region and a critical hotspot for *Impatiens* distribution (Qin et al. 2023). However, its rugged terrain, marked by high mountains and steep slopes, has only recently seen improvements in transportation, leaving many areas unexplored. Furthermore, *Impatiens* species exhibit a distinct narrow‐range distribution pattern, yet cross‐regional research remains scarce. Coupled with the unclear interspecific relationships of early‐described species, these factors have compounded existing taxonomic challenges.

During our survey of *Impatiens* species in the Yunnan‐Guizhou Plateau region, we discovered two distinct populations in Xishui County and Nayong County. The Xishui population inhabits the Danxia landform at the transition from the Yunnan‐Guizhou Plateau to the Sichuan Basin, while the Nayong population is found in the karst landform of the Wumeng Mountains. The only discernible difference between them is that the flowers of the Xishui population are yellow, whereas those of the Nayong population are purplish red. Both populations are characterized by a broadly funnelform lower sepal with violet striations and a nearly straight spur; additionally, they exhibit a taller stature, thicker stems, and more profuse branching, which clearly distinguish them from the previously reported *Impatiens* species in this region.

The morphology of reproductive organs is directly related to species propagation and gene transmission. Subjected to stronger natural selection pressure, these organs exhibit more stable evolutionary rates and narrower variation ranges. In contrast, vegetative organs are prone to considerable phenotypic variations in response to differences in ecological conditions. In taxonomic studies of *Impatiens*, floral morphology and capsule shape have greater taxonomic significance than leaf shape. Therefore, to clarify the taxonomic status of this species, we selected floral and fruit morphological traits as the core diagnostic criteria and focused on searching for congeneric species with similar morphological characteristics and adjacent type localities within the genus *Impatiens*. Through comparison, we ultimately identified that this species is closely related to the following eight species also distributed in the Yunnan‐Guizhou Plateau: *I. piufanensis* and its variety, *I. oxyanthera*, *I. forrestii*, *I. sunii*, *I. commelinoides*, *I. tienchuanensis*, and *I. fanjingshanica*. We also tentatively hypothesized that it has a closer phylogenetic relationship with *I. piufanensis*, which shares a proximate type locality.

All these species belong to sect. *Impatiens* of the genus *Impatiens* and are widely distributed in southwestern China. According to previous studies, these species are closely related phylogenetically. Field observations have also indicated that some of them are difficult to distinguish based solely on macromorphological traits, suggesting potential taxonomic ambiguities. To clarify the interspecific relationships among them, this study conducts a comparative and phylogenetic analysis of the chloroplast genomes of two populations of this new taxonomic group, seven closely related species, and one infraspecific unit (*I. piufanensis* var. *villosa*). Our goal is to leverage the variations in chloroplast genome sequences to confirm that the newly collected specimens represent an undescribed species and to resolve some of the taxonomic uncertainties surrounding these species.

## 2 Materials and Methods

### 2.1 Morphological Research

We conducted an extensive review of literature and publications related to Chinese *Impatiens* species, including “*Flora Reipublicae Popularis Sinica*” (Volume 47) (Chen 2001) and “Flora of China” (Vol. 12) (Chen et al. 2007). The aim was to gather detailed information such as the protologue, type specimen data, synonymy, geographical distributions, and any additional notes within the protologues for each scientific name. Key resources for accessing the protologues, such as Tropicos (http://www.tropicos.org), IPNI (http://www.ipni.org), and BHL (https://www.biodiversitylibrary.org/), were thoroughly searched. We ensured that all Latin scientific names strictly adhered to the records in the IPNI database.

We also consulted specimens of relevant *Impatiens* species in major domestic and international herbaria (e.g., K, E, HIB, PE, WU). A detailed examination of the type specimens was carried out, including comparison with collection information and a thorough observation of the morphological characteristics of the plants on the specimens. Key characteristics observed, measured, recorded, and verified included plant morphology, pubescence, leaf shape and color, bracts, lateral sepals, distal lobes, lateral united petals, and lower sepals. Based on the recorded distribution sites, extensive field investigations were conducted and high‐definition color images of the plants were captured. Furthermore, supplemented by data from original literature records and field observations, we compared the morphological characteristics of the new species with those of related congeners. It should be noted that the morphological comparisons in this study were based on multi‐population field surveys and herbarium specimen examinations, which can fully reflect the morphological variation characteristics of the species. However, species identification of *Impatiens* primarily relies on floral morphology. Coupled with their pronounced narrow‐endemic distribution pattern and the fact that their habitats are mostly wet and slippery sites such as forests, streams, and gorges, wild populations are difficult to survey or access for sampling. Meanwhile, to minimize the impact on small populations, only a single sample was collected per species for genomic sequencing. The voucher specimens have been deposited in the Herbarium of Guizhou University (GZAC).

### 2.2 Sampling, DNA Extraction, and Sequencing

Samples from nine distinct species were collected from their respective type localities or surrounding areas. Fresh, healthy leaves were immediately collected and preserved using silica gel desiccation. Total DNA was extracted from each of the nine samples using the CTAB method (Table 1). The quality of the extracted DNA was assessed using 1% agarose gel electrophoresis. Additionally, DNA purity was measured with a fluorometer‐based nucleic acid quantifier (Qubit). Only DNA samples that passed these quality checks were sent for sequencing. Sequencing was performed by Sangon Biotech (Shanghai) Co. Ltd. on the Illumina high‐throughput sequencing platform. After sequencing, the raw data were trimmed and filtered using FastQ (Chen et al. 2018). The processed data were then aligned with published reference sequences of *I*. sect. *Impatiens* species. De novo assembly was performed using SOAPdenovo 2 (Luo et al. 2012) and NOVOPlasty (Dierckxsens et al. 2017) to generate circular chloroplast (cp) gene maps. We evaluated and statistically analyzed the genomic sequencing data, extracted the sequencing depth and coverage information of each sample, and determined that the minimum sequencing depth was 1254.0× and the average sequencing depth was 2238.3×, which fully met the requirements for chloroplast genome assembly and subsequent analyses. In addition, the genomic integrity evaluated via BUSCO was rated as Complete.

**TABLE 1:** Information on 10 chloroplast genome samples.

Number	Species	Location	Collection number	GenBank accession number
1	*Impatiens oxyanthera* Hook. f.	Mount Emei, Sichuan, China	EMS‐2023090301(GZAC)	PQ877657
2	*Impatiens piufanensis* Hook. f.	Duyun City, Guizhou, China	DPS‐2023052001(GZAC)	PQ877658
3	*Impatiens forrestii* Hook. f. ex W. W. Sm.	Dali City, Yunnan, China	LPS‐2023101401(GZAC)	PQ877659
4	*Impatiens piufanensis* var. *villosa* G. W. Hu, S. X. Ding & S. Peng	Xianfeng County, Hubei, China	PBY‐399	PQ877660
5	*Impatiens sunii* S. H. Huang	Panzhou City, Guizhou, China	LPS‐2021100301(GZAC)	PQ877661
6	*Impatiens fanjingshanica* Y. L. Chen	Jiangkou County, Guizhou, China	FJS‐2020102401(GZAC)	PQ877662
7	*Impatiens commelinoides* Hand.‐Mazz.	Yongxing County, Hunan, China	YTH‐1	PQ877663
8	*Impatiens tienchuanensis* Y. L. Chen^a^	Tianquan County, Sichuan, China	—	OR135417
9	*Impatiens xishuiensis* X.X. Bai (flower yellow)	Xishui County, Guizhou, China	XS 20221021	PQ877664
10	*Impatiens xishuiensis* X.X. Bai (flower purple)	Nayong County, Guizhou, China	GTB 20211003	PQ877665

^a^ Download for NCBI.

### 2.3 Chloroplast Genome Annotation

For chloroplast genome annotation, *Impatiens macrovexilla* var. *yaoshanensis* (NCBI accession: OK310516) and *I. fanjingshanica* (NCBI accession: MW411294.1) were used as reference sequences. The unannotated species were first annotated using the online tool GeSeq (https://chlorobox.mpimp‐golm.mpg.de/geseq.html) (Tillich et al. 2017). Sequences were then realigned with the references, and any discrepancies were manually adjusted using Geneious R9.0.2 (Kearse et al. 2012) The final chloroplast genome maps were generated using the online program OGDRAW v1.3.1 (Lohse et al. 2013) (http://ogdraw.mpimp‐golm.mp‐g.de/).

### 2.4 Comparative Genomics

Nine genomes were newly sequenced, and 1 (*I. tienchuanensis*) was obtained from NCBI, for a total of 10 analyzed species. To examine the contraction or expansion of the inverted repeat (IR) regions within the chloroplast genes, CPJSdraw (Li et al. 2023) was employed to compare and visualize the IR boundaries, including the LSC/IRB, IRB/SSC, SSC/IRA, and IRA/LSC boundaries. The mVISTA online tool (Frazer et al. 2004) (https://genome.lbl.gov/vista/mvista/submit.shtml) was used for a comprehensive comparison of the chloroplast genomes of the 10 *Impatiens* species. Sequence alignment was performed with MAFFT v.7.5.1.1 (Katoh and Standley 2013) (https://mafft.cbrc.jp/alignment/server/). The level of nucleic acid polymorphism (Pi) was calculated using DnaSP v.6.12.03 (Rozas et al. 2017) to identify highly variable regions across the chloroplast genomes. Coding sequence (CDS) segments longer than 300 base pairs were filtered out to determine the preference for specific synonymous codons. The CodonW 1.4.2 software (Shields and Sharp 1987) was used to perform a relative synonymous codon usage (RSCU) analysis. Additionally, DNASTAR Lasergene was used to conduct a homology analysis of the 10 genome sequences.

### 2.5 Phylogenetic Analysis

23 chloroplast genome sequences, including the outgroup *Hydrocera triflora*, were downloaded from NCBI. Along with the nine chloroplast genomes obtained in this study, a total of 32 sequences were used (Table S1). The CDS sequences of each sample were separately extracted and aligned using MAFFT v.7.505 (Katoh and Standley 2013), followed by multiple sequence alignment and trimming with the retention of missing data. ModelFinder was employed to determine the optimal‐fit evolutionary models and partitioning schemes for the two datasets (i.e., the complete chloroplast genomes and CDS sequences). A phylogenetic tree was constructed using the maximum likelihood (ML) and Bayesian inference (BI) methods in Phylosuite v.1.2.3 (Zhang et al. 2020; Kalyaanamoorthy et al. 2017). The ML analyses were performed in IQ‐TREE (Minh et al. 2020), with branch support evaluated via 1000 standard bootstrap replicates (BS). Bayesian inference (BI) was implemented in MrBayes (Ronquist et al. 2012), with the analysis run for 2,000,000 generations (4 chains, 2 independent runs) to verify the consistency of results. Trees were sampled every 1000 generations, with the first 25% discarded as burn‐in. Convergence was confirmed by effective sample sizes (ESS) > 1000 (Tracer v.1.7.2; Rambaut et al. 2018) and potential scale reduction factors (PSRF) = 1.00. The reliability of each branch was assessed by calculating its posterior probability (PP). Finally, iTOL v6 (Ivica and Peer 2024) (https://itol.embl.de/) was used for visualizing the phylogenetic tree.

## 3 Results

Based on morphological comparisons and subsequent molecular analyses of chloroplast genomes, we determined that these two flower‐color variant populations belong to the same species; moreover, this species exhibits distinct differences from all previously published taxa. On this basis, we recognize it as a new species, and the existence of synonyms among closely related species has been confirmed. To facilitate the interpretation of the study results, this paper will first provide a formal description and documentation of the new species.

**Morphological characterization of new taxa**

**FIGURE 1:**
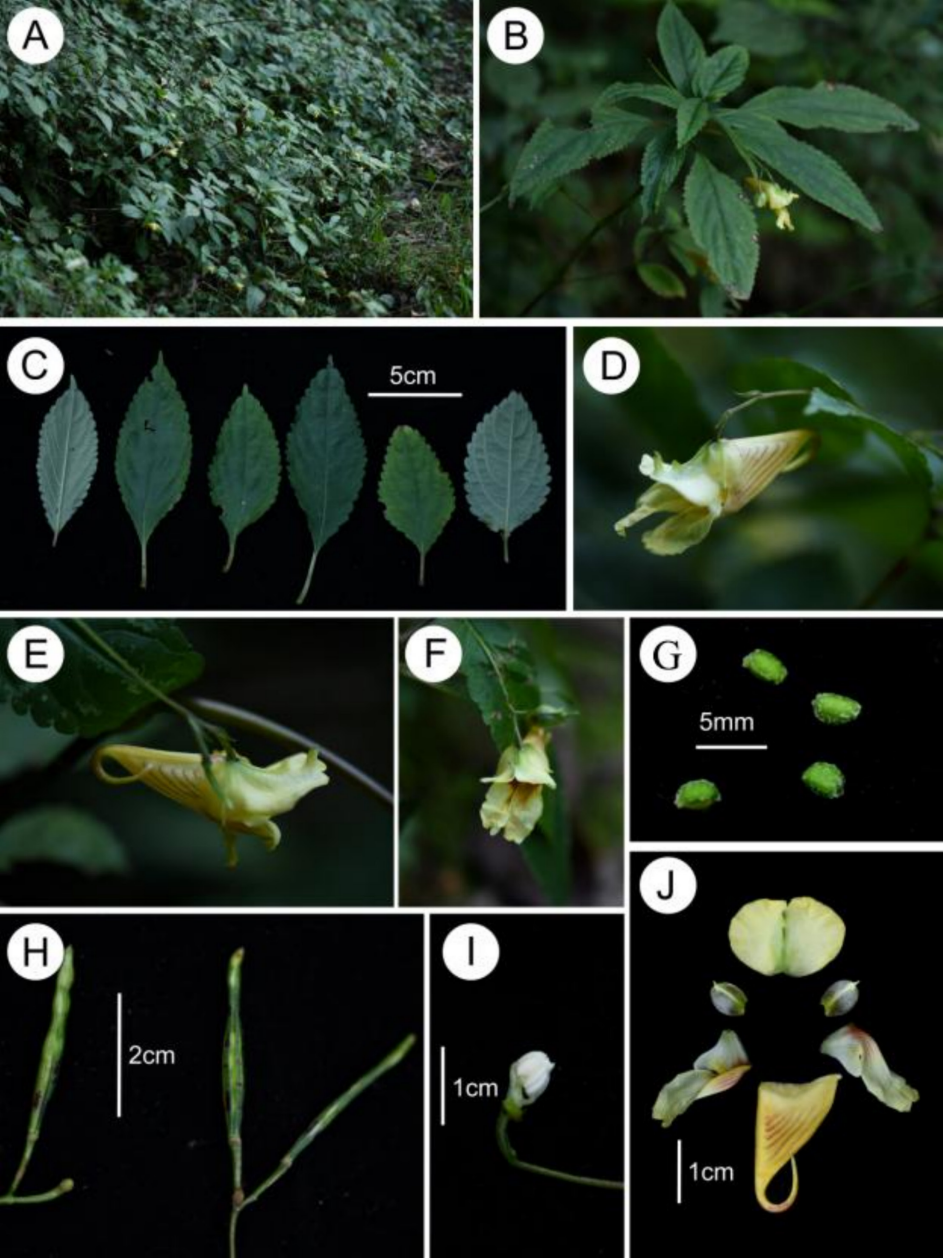
*Impatiens xishuiensis* (Xishui population). (A) habit, (B) plant, (C) leaves, (D, E) flower in lateral view, (F) flower in face view, (G) seeds, (H) fruit, (I) anther, (J) flower dissected.

**FIGURE 2:**
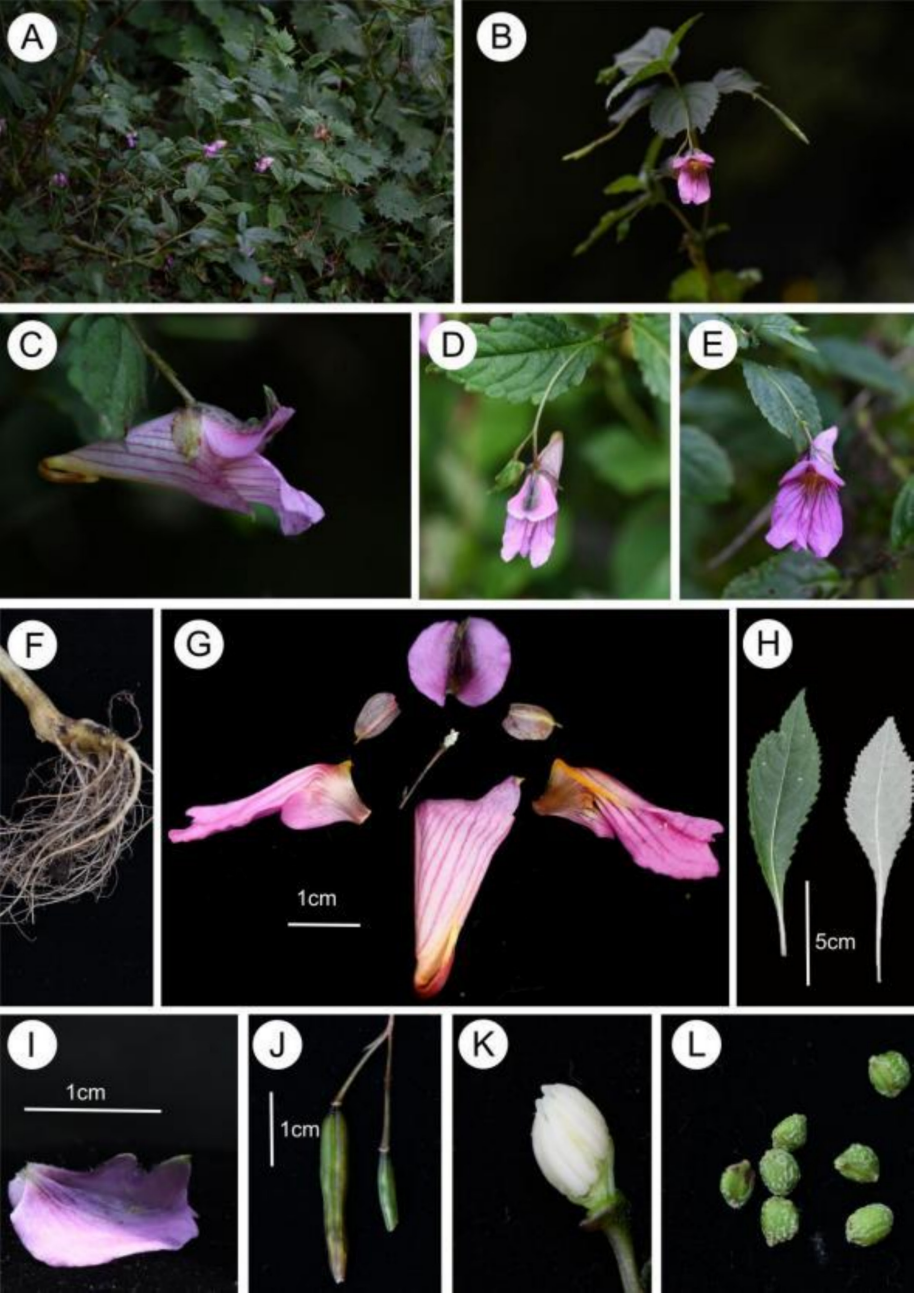
*Impatiens xishuiensis* (Nayong population). (A) Habit, (B) plant, (C) flower in lateral view, (D, E) flower in face view, (F) root, (G) flower dissected, (H) leaves, (I) upper petal, (J) fruit, (K) anther, (L) seeds.

**FIGURE 3:**
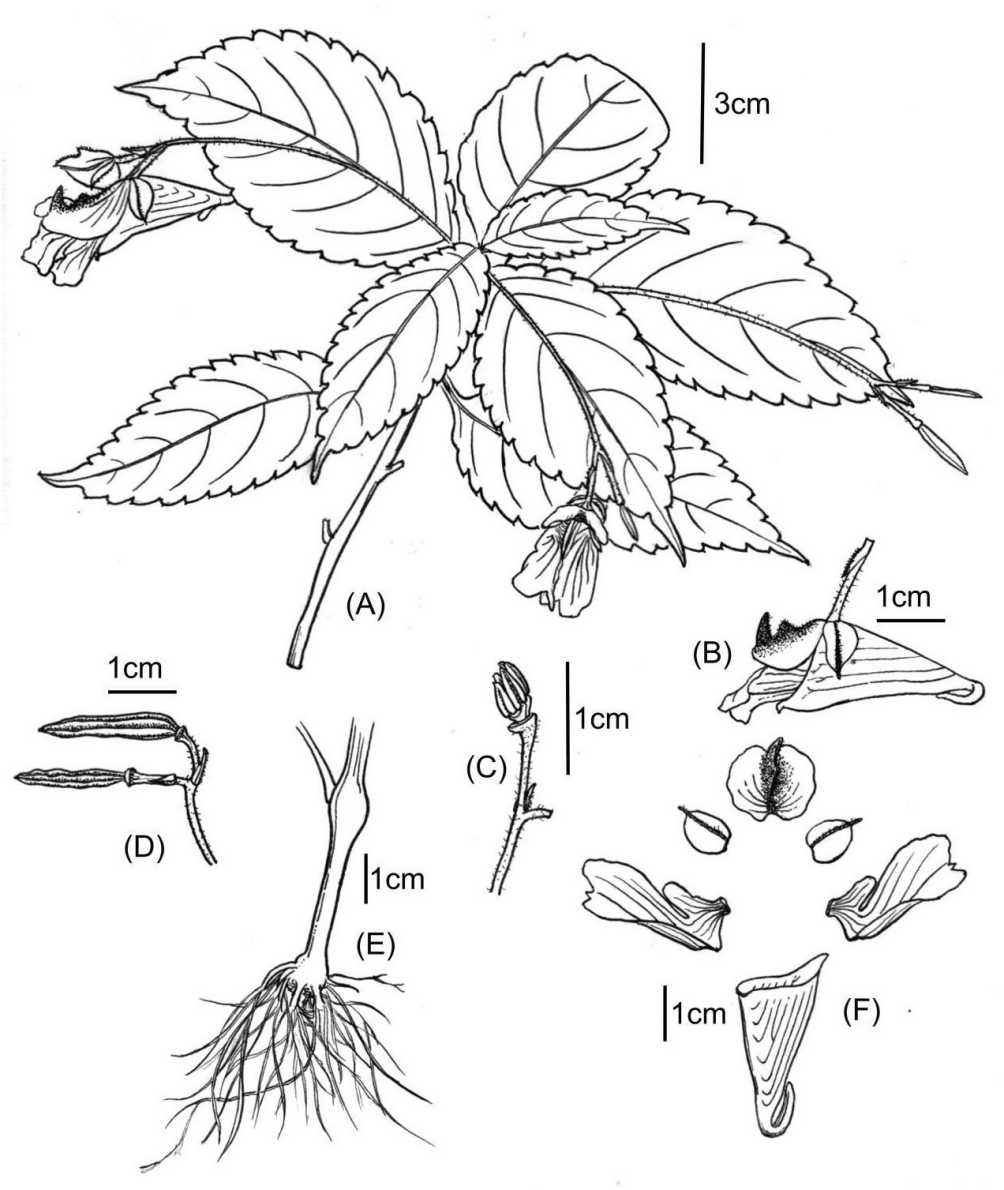
*Impatiens xishuiensis*. (A) Plant, (B) flower in lateral view, (C) anther, (D) fruit, (E) root, (F) flower dissected (drawings by Yi Chen).

***Impatiens xishuiensis* X.X.Bai, sp. nov**. Figures 1, 2, 3

*Type Locality*: Guizhou Xishui National Nature Reserve, Xishui County, Guizhou Province, China. The plant was discovered in the understory of the forest at coordinates 105°53′17.62″ E, 28°9′1.13″ N, altitude 1180 m. Collected on October 21, 2022, Bai Xinxiang et al. *XS 20221021* (*holotype*: GZAC!, isotype: GZAC!). Davidia involucrata Nature Reserve, Nayong County, Bijie City, Guizhou Province, China. Found along the roadside in the forest understory, at 26°44′45.24″ N, 105°15′32.73″ E, altitude 1980 m. Collected on October 3, 2021, Bai Xinxiang et al. *GTB 20211003*.

*Character Description*: Annual herb, 60–120 cm tall. Stems erect, glabrous, branched, with swollen nodes in lower part. with few supporting roots and many fibrous roots, Leaves alternate, petiolate, membranous, oval or oblong‐lanceolate, lamina 8.5–11.5 × 3.5–5 cm, lateral veins 4–8 pairs. The upper surface is dark green, with minute spinules along the veins, and the lower surface is gray—green, glabrous. base cuneate, margin crenate, apex acuminate, the margin is crenate—serrate. In florescences axillary, 2‐flowered, puberulent, 5–9 cm, shorter than leaves or as long as leaves, with 2 flowers. Pedicels 1–2 cm long, bracteate at base or middle, bracts narrowly lanceolate, abaxial mid‐vein puberulent. Flowers are yellow or purple, about 3 cm long. Lateral sepals 2, ovate, 1.3–1.4 × 1.0–1.1 cm, translucent, with a distinct mid—vein, apex apiculate, inequilateral. Lower sepal broadly funnelform, ca. 2.5 cm long, violet striate, mouth obliquely upwards, base gradually narrowed into a hooked spur, spur ca. 1.2 mm long. Dorsal petal reniform, 1.2–1.3 × 1.6–1.8 cm, abaxialmid‐vein thickened, green, with a dorsal crest, apex long rostellate, puberulent. Lateral united petals sessile, 2.2–2.5 cm long, violet striate, 2‐lobed, the basal lobes oval, distal lobes large, oblong, auricle inflexed, emarginate at apex. Stamens 5, filaments linear, anther pointed, ovary linear, apex beak pointed. Capsule clavate, seeds many, verrucosa.

*Phenology*: Flowering from September to October; fruiting from November to December.

Etymology: The specific epithet “*xishuiensis*” refers to the type locality of this species, Guizhou Xishui National Nature Reserve in Xishui County.

*Distribution*: *I. xishuiensis* was discovered at the Linjiang Management Station of Xishui National Nature Reserve in Xishui County, Guizhou Province. In addition, it is also distributed in Davidia involucrata Nature Reserve of Nayong County and Jiulong Mountain of Dafang County, both in Bijie City. This species is concentrated in the forest understory or along roadsides in valleys, with a relatively large population size.

*Conservation Status*: Currently, *I. xishuiensis* has been found distributed in Xishui National Nature Reserve of Zunyi City, Guizhou Province, as well as Davidia involucrata Nature Reserve of Nayong County and Jiulong Mountain of Dafang County, both in Bijie City. The population size is considerable. The ecological environment within these reserves is well preserved, free of road construction, and minimally disturbed by human activities. According to the categories and criteria of the IUCN Red List (IUCN, 2019), its conservation status is assessed as Least Concern (LC).

*Annotation*: At present, only a few populations have been recorded in Xishui and Nayong, Guizhou Province. However, considering that there are numerous sites with similar habitats in Guizhou—including areas characterized by both karst and Danxia landforms—coupled with the lack of systematic surveys in adjacent regions and the extensive distribution of its closely related species, this species may have a far broader distribution range, indicating that it has the potential to be a widespread taxon.

### 3.1 Chloroplast Genomes of the New Species

**FIGURE 4:**
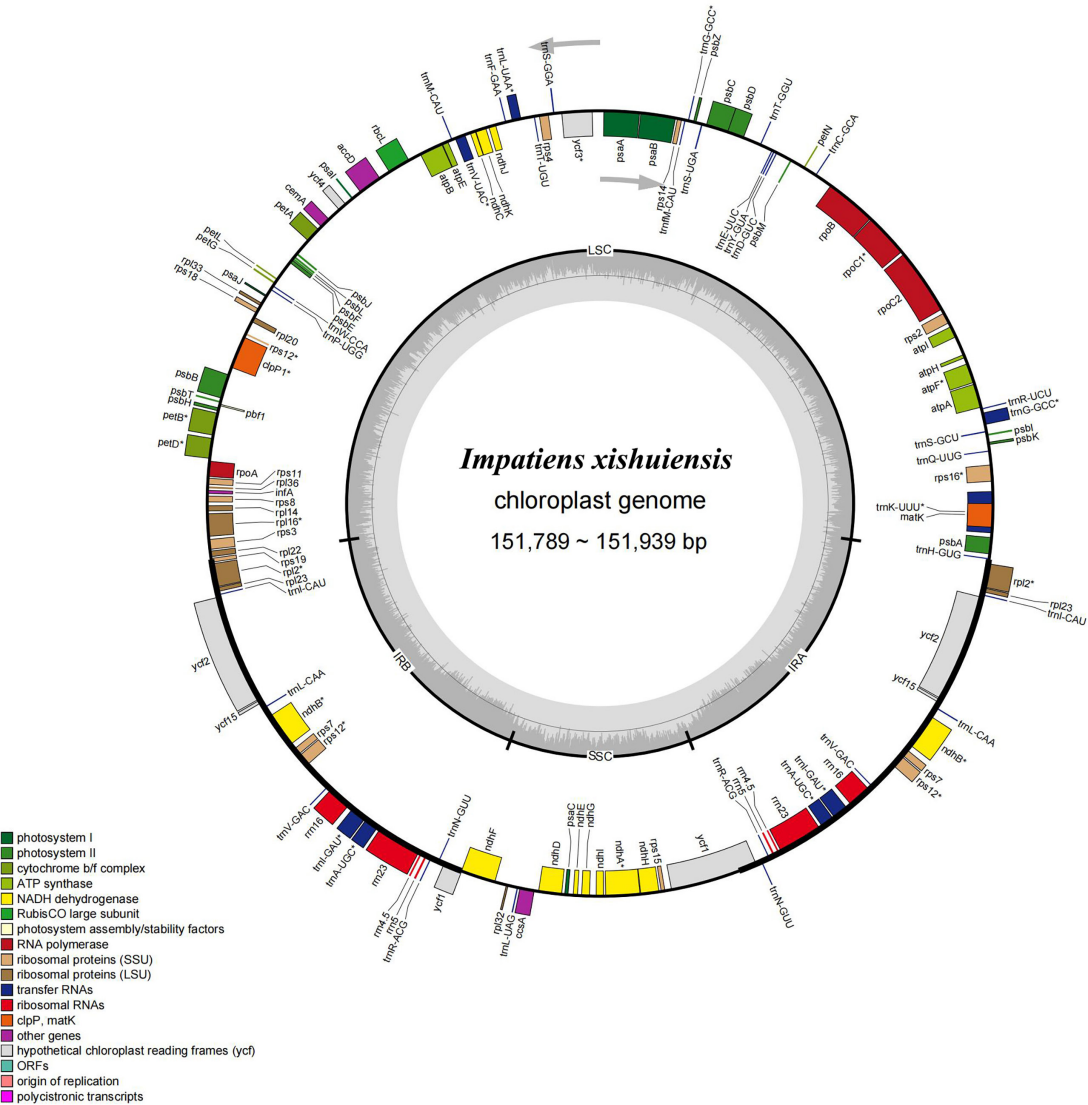
Chloroplast genome map of *Impatiens xishuiensis* (Genes located inside the circle are transcribed in a clockwise direction, while those on the outside of the circle are transcribed in a counterclockwise direction).

The chloroplast genomes of the newly discovered *Impatiens* species exhibit a typical quadripartite structure (Figure 4). The chloroplast genome length of the yellow‐flowered population of *I. xishuiensis* in Xishui is 151,939 bp, while that of the purple‐flowered population in Nayong is 151,789 bp. These genomes consist of a large single‐copy region (LSC) (83,796–82,905 bp), a small single‐copy region (SSC) (17,459–17,502 bp), and a pair of inverted repeat regions (IRs) (51,766–51,767 bp). There are slight variations in the GC content of different regions within the chloroplast genome (Table 2). The overall GC content ranges from 36.84% to 36.87%. Additionally, the GC content at the first, second, and third codon positions, from lowest to highest, is as follows: third position (28.92%–28.93%), second position (37.53%–37.55%), and first position (44.77%–45.78%).

**TABLE 2:** Structure of the complete cp genomes of the *Impatiens xishuiensis*.

	*I. xishuiensis*‐1	*I. xishuiensis*‐2
Genome size (bp)	151,939	151,789
GC (%)	36.84	36.87
LSC size (bp)	82,905	82,796
SSC size (bp)	17,502	17,459
IRs size (bp)	25,766	25,767
GC of LSC (%)	34.53	34.55
GC of SSC (%)	29.38	29.39
GC of IR (%)	43.08	43.11
GC of CDSs (%)	37.08	37.08
1st position GC (%)	44.78	44.77
2nd position GC (%)	37.53	37.55
3rd position GC (%)	28.93	28.92
Length of CDSs	80,118	80,112
Number of CDSs	88	88

Each chloroplast genome contains 113 genes (Figure 4, Table S2). These genes can be categorized into three groups: (1) Transcription‐related and RNA genes. Including 4 transcription‐related genes (*rpoA*, *rpoB*, *rpoC1*, *rpoC2*), 21 ribosomal proteins, 4 ribosomal RNAs (*rrn4.5*, *rrn5*, *rrn16*, *rrn23*), and 29 transfer RNAs; (2) Photosynthesis‐related genes: 47 genes related to photosynthesis, found in the Rubisco, ATP synthase, photosystem I, cytochrome b/f complex, photosystem II, cytochrome c synthesis, and NADPH dehydrogenase groups; (3) 8 other genes.

### 3.2 Comparative Genomic Analysis, Nucleotide Polymorphism and Condon Ussage

**FIGURE 5:**
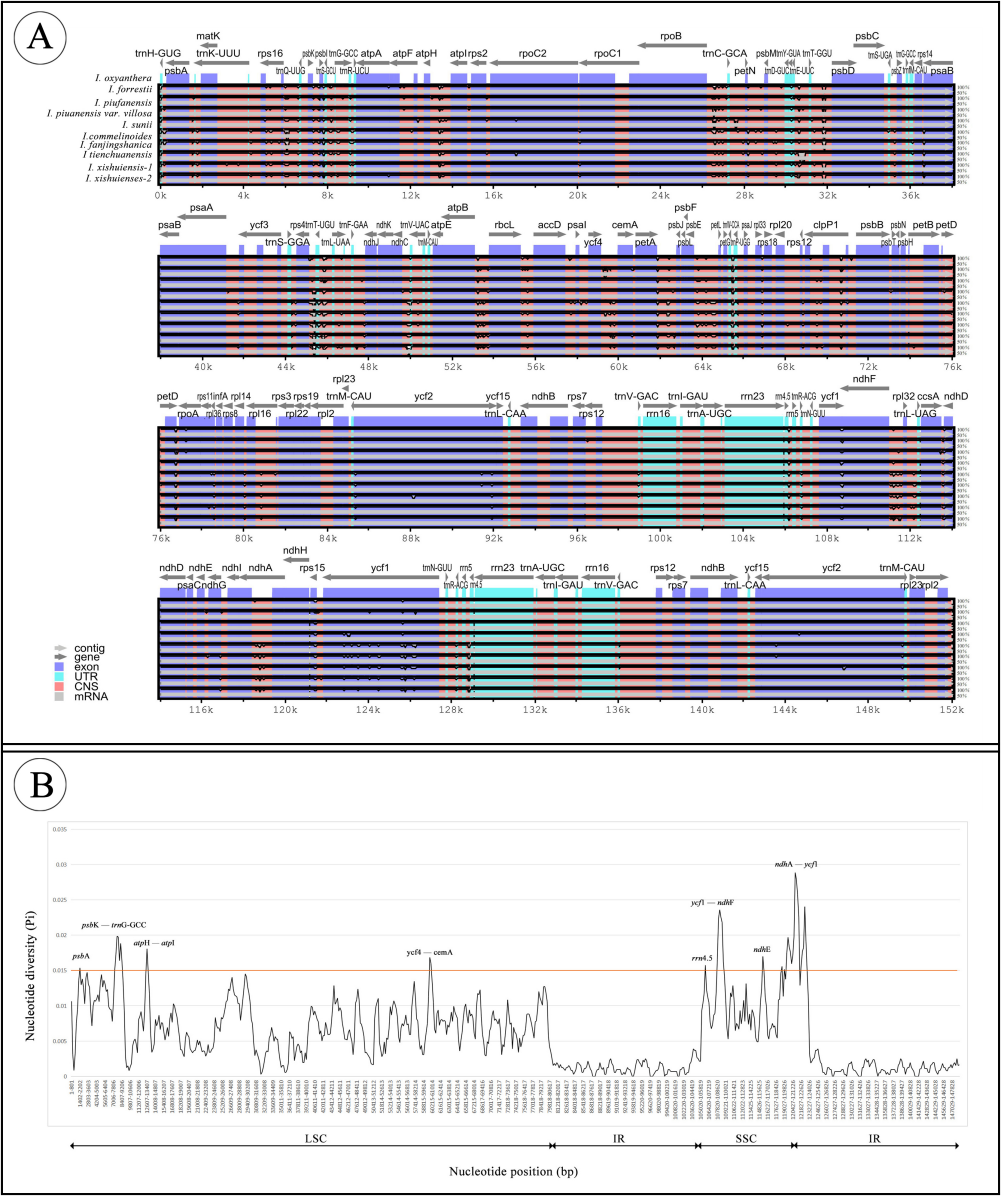
Genome variability analysis. (A) mVISTA genome comparison (The *x*‐axis represents the genome coordinates, and the *y*‐axis represents the percentage of identity [from 50% to 100%]). The arrows indicate the direction of each gene. CNS, non‐coding sequence; UTR, untranslated region. (B) Nucleotide diversity (Pi) values of the chloroplast genomes of 10 *Impatiens* species.

Comparative sequence analysis indicates that the four gene regions across the 10 chloroplast genomes show minimal differences. The number and sequence of genes in the IR region are highly conserved, with smaller variations compared to the LSC and SSC regions. Sequence variation is higher in the non‐coding regions than in the coding regions (Figure 5). The DNA nucleotide polymorphism (Pi) analysis (Figure 5) reveals that the nucleotide diversity (Pi) among the 10 species ranges from 0 to 0.02885, with an average value of 0.00588. Using a Pi threshold > 0.015, several highly variable regions were identified. Specifically, the *ycf1‐ndhF* and *ndhA‐ycf1* regions exhibit a Pi value > 0.015, with the Pi value of the *ndhA‐ycf1* region exceeding 0.025, suggesting that this region holds significant potential as a molecular marker. Additionally, the Pi value in the IR region is substantially lower than that in the SSC and LSC regions, indicating that mutations are far more frequent in the LSC and SSC regions compared to the IR region.

Statistical analysis was performed based on the relative synonymous codon usage (RSCU; Table S3; Figure S1). A total of 64 distinct RSCU values were obtained. Among these values, 30 codons had an RSCU value > 1.00 and were identified as high‐frequency codons. The codons encoding methionine (AUG) and tryptophan (UGG) showed no codon usage bias. Except for the three stop codons (UAA, UAG, and UGA), the remaining 61 codons encoded the 20 standard amino acids.

### 3.3 IR Boundaries

**FIGURE 6:**
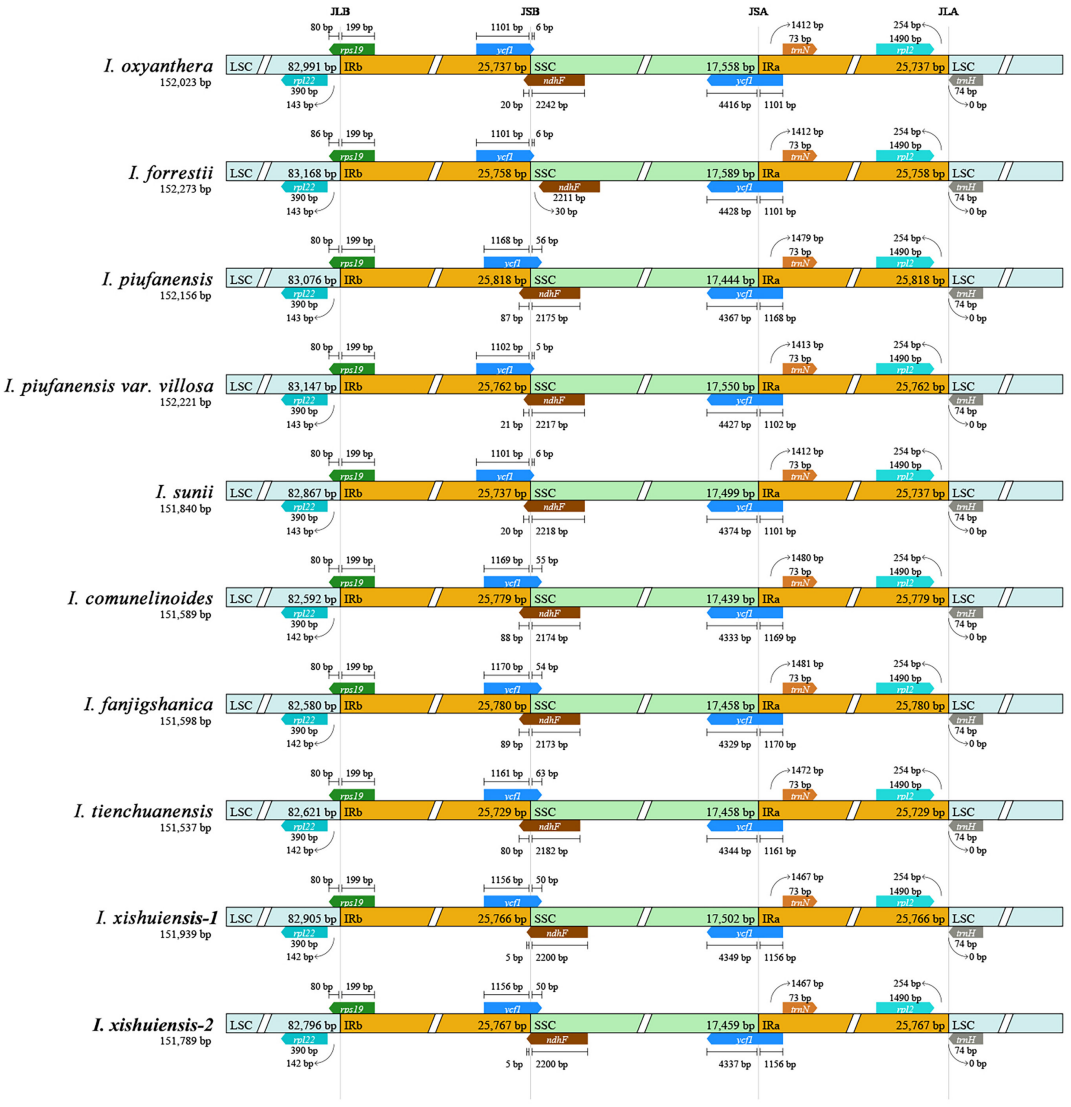
Analysis of the IR region boundaries of chloroplast genomes of 10 *Impatiens* species. The gray arrows indicate the transcription direction of genes. IR, inverted repeat region; JLB, junction site between LSC and IRb region; LSC, large single‐copy region; SSC, small single‐copy region.

To investigate the expansion and contraction of the IR boundaries, we compared the chloroplast genomes of nine species. The results revealed the gene structures at the four junctions of the gene regions (Figure 6).

The IRB‐LSC junction (JLB) is located within the *rps19* gene, and the lengths of *rps19* in the IRB and LSC regions are identical across all 10 samples. In *I. oxyanthera*, *I. piufanensis*, *I. forrestii*, *I. piufanensis* var. *villosa*, and *I. sunii*, the distance from *rpl22* in the LSC region to IRB is 143 bp, while for other samples it is 142 bp.

The IRB‐SSC junction (JSB) is located in *ycf1*. The n*dhF* gene is absent from the IR region in *I. forrestii*, while it is present in the IR region in the other species. The amplification lengths of the *ycf1* and *ndhF* genes also vary. Two populations of *I. xishuiensis* show identical gene amplification and contraction.

The IRA‐SSC junction (JSA) is located in another segment of the *ycf1* gene that spans both the IRA and SSC regions. Its length in the SSC region ranges from 4329 to 4428 bp. The distribution of *ycf1* and *trnN* in the IRA region is consistent for *I. xishuiensis*, *I. oxyanthera*, *I. forrestii*, and *I. sunii*. There is a 1‐bp difference between *I. piufanensis* var. *villosa* and these species, and a similar difference is observed between *I. fanjingshanica* and *I. commelinoides*.

The IRA‐LSC junction (JLA) is located at the start of the *trnH* gene, where no significant expansion or contraction of the IR region was observed.

### 3.4 Phylogenetic Positions of 10 *Impatiens* Species

**FIGURE 7:**
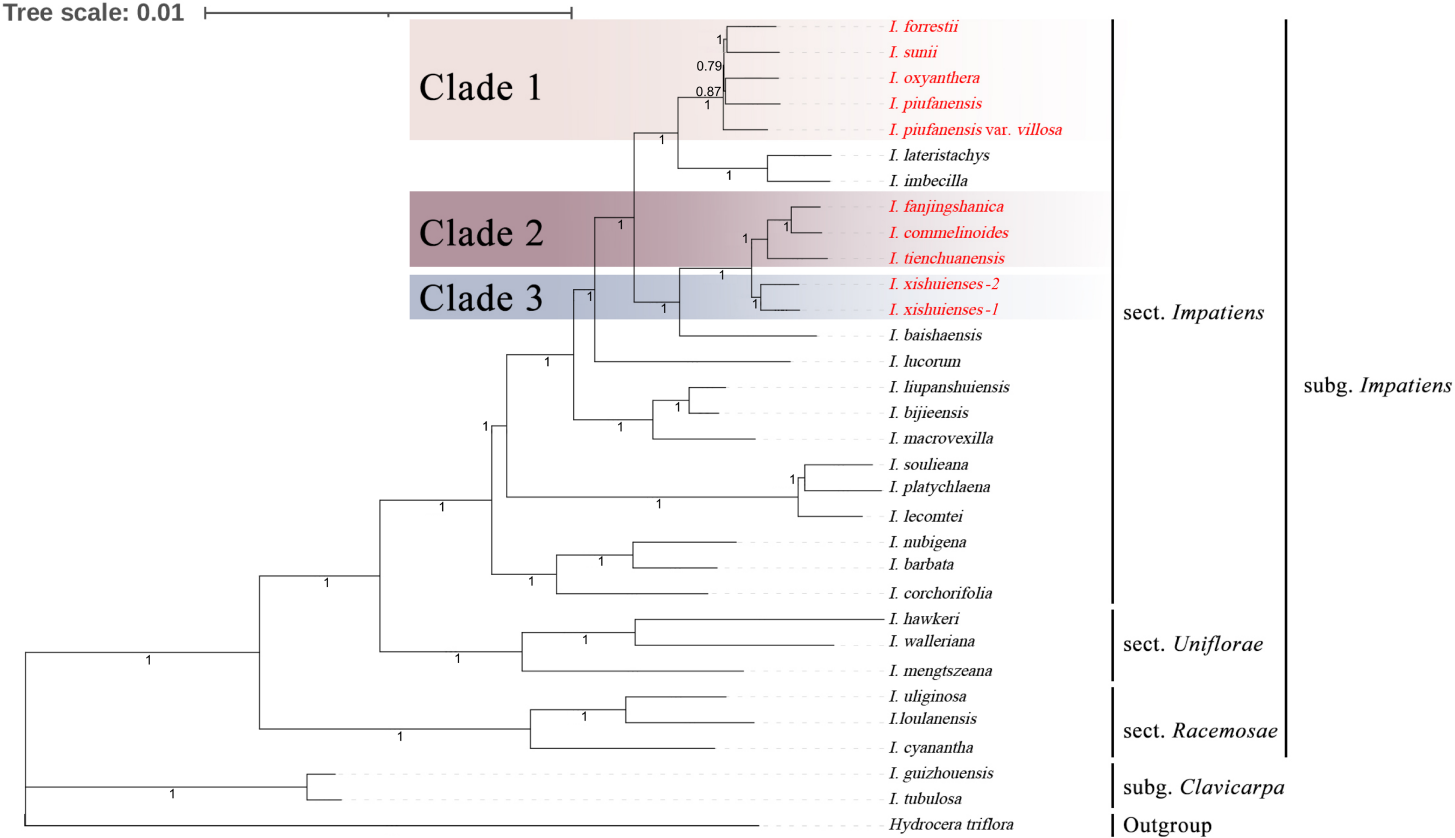
Bayesian inference (BI) tree based on the complete chloroplast genome sequences. Posterior probability (PP) values are indicated at the corresponding branches.

**FIGURE 8:**
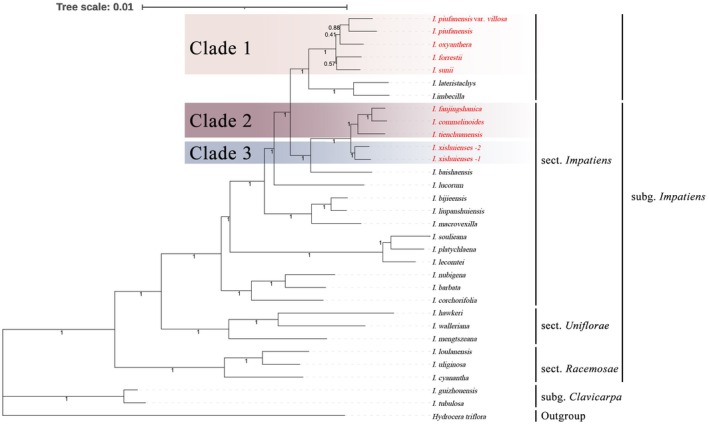
Maximum likelihood (BI) tree based on the CDS sequences of 32 species. (Posterior probability (PP) values are indicated at the corresponding branches.)

A phylogenetic analysis was conducted using complete chloroplast genomes to explore the evolutionary relationships among the 10 *Impatiens* species. These species, with documented distributions in China, represent two subgenera and three sections. *Hydrocera triflora*, a member of the Balsaminaceae family, was used as the outgroup. For the phylogenetic tree constructed using the complete chloroplast genome sequences, the optimal substitution model selected for the Maximum‐Likelihood (ML) method was GTR + F + I + I + R6, and that for Bayesian Inference (BI) was GTR + F + I + G4. For the phylogenetic tree constructed using CDS sequences, the evolutionary rates varied among individual genes; the detailed partitioning schemes for both ML and BI analyses are documented in Tables S4 and S5. Both the phylogenetic trees based on complete chloroplast genomes and those derived solely from coding DNA sequences (CDS) showed similar topologies, with very high branch support values (Figures 7 and 8). Therefore, only the Bayesian phylogenetic tree is presented here, while the Maximum Likelihood (ML) tree can be found in Figures S2 and S3.

The results show that all 10 species belong to *I*. sect. *Impatiens*. *I. oxyanthera*, *I. piufanensis*, *I. piufanensis* var. *villosa*, *I. sunii* and *I. forrestii*, were stably clustered into a single clade (Clade 1) in the phylogenetic analyses. The monophyly of this clade was strongly supported by both phylogenetic trees (PP = 1.00), indicating that these five taxa share a close common evolutionary origin. Notably, within this clade, the phylogenetic tree constructed based on complete chloroplast genome sequences and that built from chloroplast coding sequences (CDS) showed overall topological consistency but exhibited minor topological differences, which were mainly reflected in the phylogenetic position of *I. piufanensis* var. *villosa*. Specifically, in the tree derived from complete chloroplast genomes, *I. piufanensis* var. *villosa* and *I. oxyanthera* were sister taxa, and the clade formed by these two taxa was further clustered with other taxa. In contrast, in the tree constructed from CDS sequences, *I. piufanensis* var. *villosa* formed a sister‐taxon relationship with its original variety, *I. piufanensis*.

It should be supplemented that *I. piufanensis* var. *villosa* was first discovered and named by Ding et al. (2022). Its early taxonomic delimitation mainly relied on sequencing results of short‐fragment DNA molecular markers (ITS, atpB‐rbcL) combined with morphological character comparisons, and the evidence from that study supported the classification of this variety and its original variety *I. piufanensis* into the same species. However, the results of the phylogenetic analysis based on complete chloroplast genomes in this study revealed that *I. piufanensis* is more closely related to *I. oxyanthera* than to *I. piufanensis* var. *villosa*, which highlights the complexity of the evolutionary relationships among species within Clade 1.

Clade 2 was formed by *I. fanjingshanica*, *I. commelinoides*, and *I. tienchuanensis*. The monophyly of this clade was also strongly supported (PP = 1.00), indicating that the evolutionary relationships among these three taxa are stable and unambiguous. In addition, *I. xishuiensis* and samples from its two distinct wild populations were resolved as an independent clade (Clade 3), whose monophyly was also strongly supported (PP = 1.00). The phylogenetic topology showed that Clade 3 is more closely related to Clade 2 in the evolutionary lineage, and the two clades together form a larger monophyletic lineage.

It should be emphasized that both the phylogenetic analysis based on complete chloroplast genome sequences and that based on CDS sequences have confirmed the independent taxonomic status of *I. xishuiensis*.

### 3.5 Nucleotide Homology of Chloroplast Genomes

**FIGURE 9:**
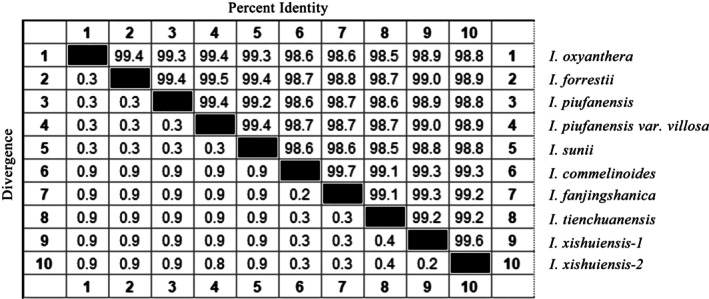
Homology analysis of 10 chloroplast genomes.

The chloroplast genome sequences provide comprehensive data that enhance the understanding of phylogenetic relationships and species identification efficiency (Niu et al. 2018; Pfanzelt et al. 2019). The nucleotide homology analysis reveals extremely high homology among the 10 *Impatiens* species, ranging from 98.6% to 99.6% (Figure 9). Specifically, the homology within Clade 1 is greater than or equal to 99.2%, while homology in Clades 2 and 3 is greater than or equal to 99.1%. Notably, the nucleotide homology among populations of *I. xishuiensis* with different flower colors reaches 99.7%.

### 3.6 Publication History and Morphological Comparison

#### 3.6.1 Publication History and Morphological Variations Among Species in Clade 1

In 1908, the botanist J. D. Hooker formally described and published *Impatiens oxyanthera* (Hooker, 1908a) based on specimens collected from Mount Emei, Sichuan Province. Meanwhile, he also published *I. piufanensis* (Hooker, 1908b) using specimens collected from Pingfa (now part of Guiding County) in Guizhou Province. According to records in *Flora of China* (Volume 47, Part 2) (Chen 2007), the lower or basal part of the stem of *I. oxyanthera is* usually swollen to form a tuberous structure, with numerous fibrous roots attached. However, these descriptions still indicate that *I. piufanensis* possesses morphological features distinguishable from *I. oxyanthera*: its pedicels bear only one flower, with bracts located at the upper part. In addition, its flowers are relatively larger, and the midrib on the dorsal side of the vexillum features a distinct rostriform protuberance below the apex. These features constitute the core diagnostic criteria for their traditional taxonomic distinction. Through the re‐examination of the type specimens of the two aforementioned *Impatiens* species, retrospective of the original literature, and field investigations at their type localities, this study found that the pedicels of *I. piufanensis* actually produce two flowers, rather than one flower as documented in the original description. This taxonomic misjudgment is speculated to result from the undeveloped state of one flower bud in the type specimen. Moreover, in different natural populations at the type localities of the two species, the dorsal crest on the distal lobes exhibits a continuous variation gradient—a gradual transition from distinctly raised to flattened and non‐raised crests. This indicates that this character does not serve as a stable diagnostic trait for distinguishing the two species.

*I. forrestii* was first published in 1915 based on specimens from Dali, Yunnan (Takedar 1915). Its distinguishing features from the aforementioned species are primarily the number of leaf veins (8–9 pairs in *I. forrestii* versus 5–6 pairs in *I. oxyanthera* and *I. piufanensis*) and the shape and size of the lateral sepals: the lateral sepals of *I. forrestii* are obliquely broadly ovate or suborbicular, ca. 8–9 mm, whereas those of *I. oxyanthera* and *I. piufanensis* are ovate, ca. 2–5 mm.

However, field investigations conducted in the Dali area of Yunnan Province have revealed that the sepal color and size of *I. forrestii* exhibit significant intraspecific variation among populations, and all such variations fall within the range of differences defined by the aforementioned traditional diagnostic characters, showing a pattern of continuous transition. More notably, within the same natural population of *I. forrestii*, the leaf vein number of some individuals is only 5–6 pairs, which overlaps with the vein number range of *I. oxyanthera* and *I. piufanensis*. Meanwhile, the leaf vein number of some specimens of *I. oxyanthera* and *I. piufanensis* also exceeds 6 pairs. The above findings indicate that leaf vein number is not a stable interspecific diagnostic character for these species; instead, it shows high plasticity among individuals and thus cannot be used as a reliable criterion for species delimitation within the genus *Impatiens*.

**FIGURE 10:**
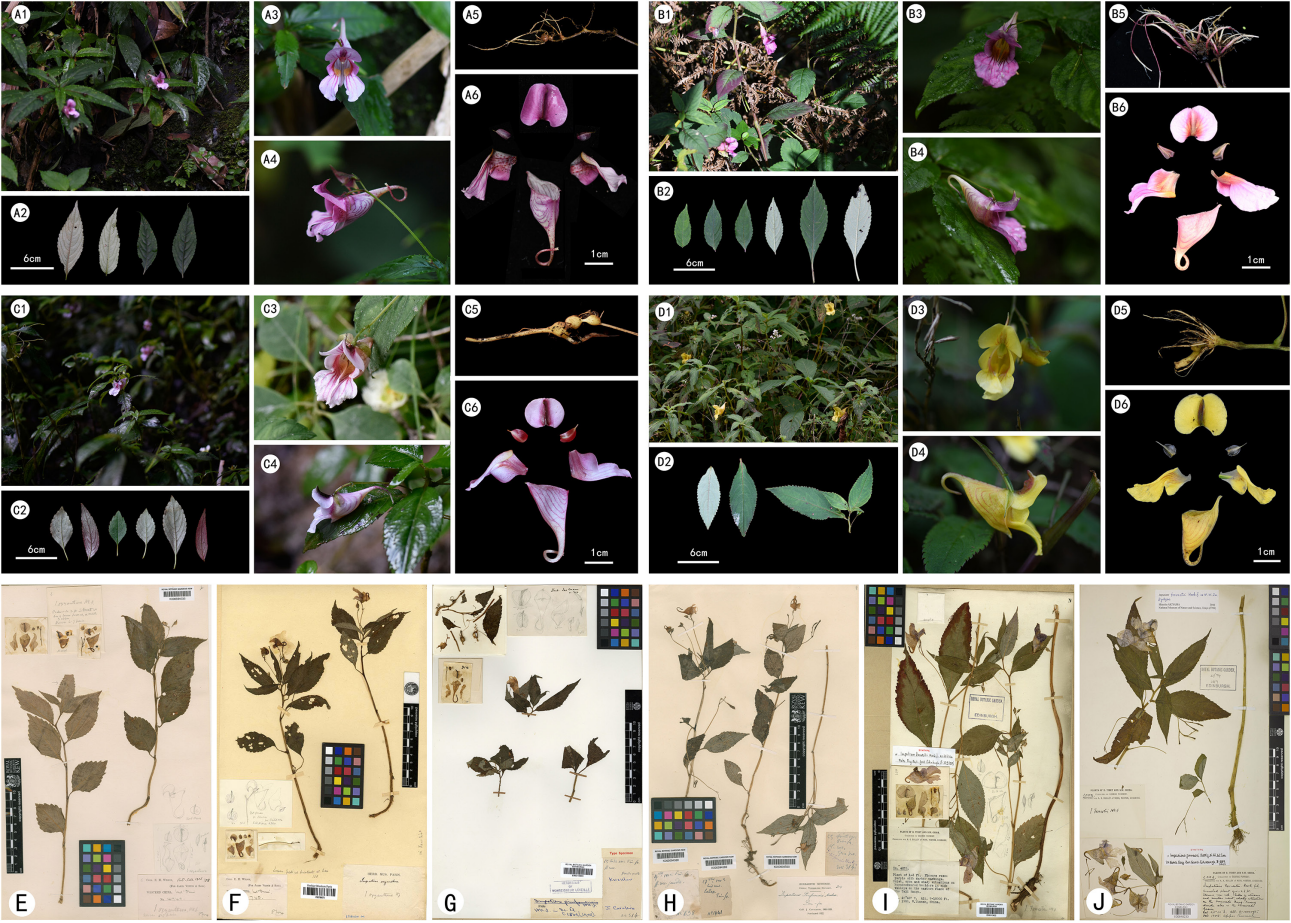
Comparative photographs of morphological characteristics of Clade1. A1–A6. I. oxyanthera. (A1. Habit, A2. Leaves, A3. Flower in face view, A4. Flower in lateral view, A5. Root, A6. Flower dissected). B1–B6. *I. forrestii* (B1. Habit, B2. leaves, B3. Flower in face view, B4. Flower in lateral view, B5. Root, B6. Flower dissected). C1–C6. *I. piufanensis* (C1. Habit, C2. leaves, C3. Flowerinfaceview, C4. Flower in lateral view, C5. Root, C6. Flower dissected). D1–D6. *I. sunii* (C1. Habit, C2. leaves, C3. Flower in face view, C4. Flower in lateral view, C5. Root, C6. Flower dissected). E–J (E–F *I.oxyanthera*, G–H *I. piufanensis*, I–J *I. forrestii*).

*Impatiens sunii* was published by Huang, He, et al. (2023); Huang, Yuan, et al. (2023). The most distinctive feature of this species from other members within Clade 1 is its yellow floral phenotype. In the protologue of *I. sunii* (Huang et al. 2003), the researchers emphasized the morphological differences between this species and *I. piufanen*sis, specifically including the following three aspects: (1) The leaf margins are adorned with triangular, coarse teeth. (2) The lateral sepals are ovate—oblong, somewhat thick, tapering to an acuminate apex. (3) The vexillum is circular, featuring a hooked beak at the tip, with a narrow wing along the midrib on the dorsal side. However, field observations conducted in this study revealed that the leaf morphology and lateral sepal characteristics of *I. sunii* do not possess distinct uniqueness, but instead show considerable overlap with those of other species in Clade 1. Moreover, the narrow wing on the dorsal midrib of its standard petals is highly similar to that of *I. piufanensis* var. *villosa*, a variety published in 2022—which has already been confirmed to be conspecific with *I. piufanensis* at the time of its publication. Collectively, the only core diagnostic criterion distinguishing *I. sunii* from other species within Clade 1 is its stable yellow floral color (Figure 10, Table 3).

**TABLE 3:** Comparison of morphological characteristics of 10 species.

Characteristics	*I. xishuiensis*	*I. piufanensis*	*I. piufanensis* var. *villosa*	*I. forrestii*	*I. oxyanthera*	*I. sunii*	*I. commelinoides*	*I. fanjingshanica*	*I. tienchuanensis*
Root	Present	Present	Present	Present	Present	Present	Absent	Absent	Absent
Stem	Erect, branched, swollen nodes	Erect, sparsely branched, basally prostrate, swollen nodes	Erect, sparsely branched, densely white villou, basally prostrate, swollen nodes	Erect, sparsely branched, swollen nodes	Erect, sparsely branched, swollen nodes	Erect, sparsely branched, swollen nodes	Prostrate, swollen nodes	Prostrate, swollen nodes	Prostrate, swollen nodes
Lateral vein	4–8 pairs	4–5 pairs	8–12 pairs	8–9 pairs	4–5 pairs	5–7 pairs	5–7 pairs	5–7 pairs	5–6 pairs
Leaf	Oval or oblong‐lanceolate	Long‐ovate or lanceolate	Ovate‐lanceolate or sub‐oval, densely white villous	Ovate‐lanceolate or sub‐oval	Ovate or ovate‐lanceolate	oval	Ovate or ovate‐rhomboid, With glands at the base	Ovate or ovate‐lanceolate, rarely sub‐rhomboid, With glands at the base	Ovate or ovate‐ lanceolate, rarely ovate‐rounded, With glands at the base
Bract	Narrowly lanceolate	Lanceolate	Lanceolate, densely white villous	Ovate‐lanceolate	Ovate	Ovate	Lanceolate or linear‐lanceolate	Ovate or ovate‐lanceolate	Linear‐lanceolate
Lateral sepals	Ovate, apex apiculate	Oval, apex apiculate	Lanceolate, apex apiculate, densely white villous	Obliquely broad‐ovate or suborbicular, apex apiculate	Oval, apex apiculate	Ovate‐oblong, apex apiculate	Broadly ovate, apex apiculate	Broadly ovate to suborbicular, apex apiculate	Broadly ovate or ovate‐rounded, apex apiculate
Flower color	Yellow or purplish red	Red	Rose	Purplish red	Purplish red	Yellow	Purple	Purple	Purple
Distal lobes	Reniform, with a dorsal crest, apex long rostellate	Round or obovate, apex acute	Suborbicular, apex long rostellatee, densely white villous	Reniform, apex acute	Round or obovate, apex acute	Round or oblate, apex acute	Round, apex acute	Round, apex acute	Round, apex acute
Distal lobes of Lateral united petals	Oblong, auricle inflexed, emarginate at apex.	Dolabriform, apex Obtuse	Dolabriform, apex Obtuse	Dolabriform, apex Obtuse	Dolabriform, apex Obtuse	Dolabriform, apex Obtuse	Dolabriform, apex Obtuse	Dolabriform, apex Obtuse	Dolabriform, apex Obtuse
Lower sepal	Broadly funnelform, violet striate, nearly straight	Funnelform, violet striate, curved	Funnelform, violet striate, curved, densely white villous	Funnelform, violetmstriate, curved	Funnelform, violet striate, curved	Funnelform, violet striate, curved	Funnelform, violet striate, curved	Funnelform, violet striate, curved	Funnelform, violet striate, curved
Seed	Verrucosa	Smooth	Smooth	—	—	—	Smooth	Smooth	Smooth

#### 3.6.2 Publication History and Morphological Differences of Species in Clade 2

*Impatiens commelinoides* was first described and published by Handel‐Mazzetti (1933). Its type locality is present—day Ningdu County, Jiangxi Province. *I. tienchuanensis*, native to Tianquan, and *I. fanjingshanica*, from Mount Fanjing in Guizhou, were published by Chen (1978, 1999) respectively. In their respective protologues, both species were mentioned to be closely related to *I. commelinoides*.

According to the protologue, *I. fanjingshanica* can be distinguished from *I. commelinoides* by the following features: (1) The bracts are ovate, and the lateral sepals have distinct pointed tips. (2) The alae are nearly stalkless, with the upper lobes being hatchet‐shaped. However, during on‐site observations of *I. fanjingshanica* at its type locality, these characteristics are completely consistent with those of *I. tienchuanensis*. With respect to the distinguishing characters reported in the aforementioned protologues, morphological transitional phenomena among different individuals have been observed in field investigations, which are specifically manifested as follows: (1) The bracts vary from ovate to ovate‐lanceolate, and the sepals may or may not have pointed tips. (2) The alae can range from stalkless to having a stalk, and the upper lobes can be from sub‐circular to broadly hatchet‐shaped.

**FIGURE 11:**
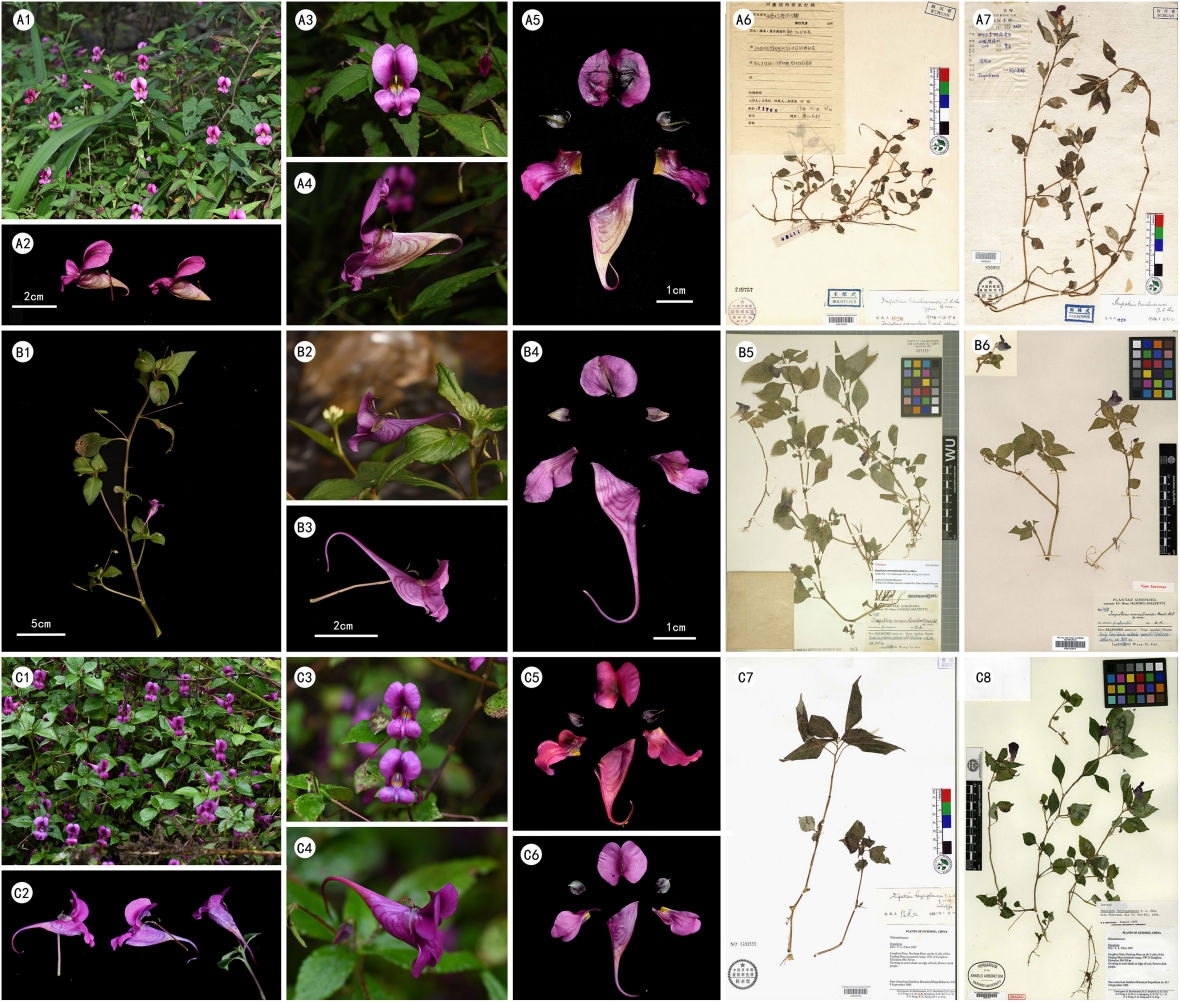
Comparative photographs of morphological characteristics of Clade2. A1–A5. *I. tienchuanensis* (A1. Habit, A2. flowers, A3. Flowerinfaceview, A4. Flower in lateral view, A5. Flower dissected A6–A7 type). B1–B6. *I. commelinoides* (B1. Plant B2–B3. Flower in lateral view B4. Flower dissected B5–B6 type. C1–C8). *I. fanjingshanica* (C1. Habit, C2. flowers, C3. Flower in face view, C4. Flower in lateral view, C5–C6. Flower dissected, C7–C8, type) (A1–A5 Takenby TaohuaYuan).

In its protologue, *I. tienchuanensis* was said to differ from the other two species in several ways: (1) The lower surface of the leaves has sparse short hairs, and the mid‐veins of the bracts and sepals are also sparsely covered with short hairs. (2) The top of the vexillum is deeply indented, its edge is slightly wavy, and there is a crest‐like protrusion below the top of the mid‐rib on the back. (3) The tip of the spur on the labellum is shallowly two‐lobed. Nevertheless, field investigations revealed that these features do not constitute substantial differences. Firstly, the description of *I. commelinoides* seems incomplete, as its leaf undersides, and the mid‐veins of its bracts and sepals also have sparse short hairs. Secondly, in the late stage of flower blooming, the vexillum of *I. tienchuanensis* slightly curls backward, which accounts for the wavy edge described. Under normal circumstances, there is no obvious difference in the vexillum among these species. Thirdly, the characteristic of the spur with a shallowly two‐lobed tip is prevalent across all 10 taxa involved in this study (Figure 11, Table 3).

### 3.7 Taxonomic Treatment

Based on the continuous c molecular phylogenetic evidence discussed above, the relevant scientific names should be tentatively treated as synonyms pending more comprehensive sampling investigations, and the taxonomic treatment is presented herein.

In the initial publication of *Impatiens oxyanthera*, no holotype was designated. Currently, three isotype specimens exist, conserved at herbaria P, A, and K. The protologue notes that the type specimens were deposited at herbaria P and K. Among them, the specimen numbered K000694033, held in herbarium K, contains more extensive floral morphological details. Hence, this specimen is designated as the lectotype.

Regarding *I. forrestii*, its original publication also lacked the designation of a holotype. There are three syntype specimens in existence. The specimen numbered E00313622 aligns more closely with the information described in the literature and encompasses complete plant morphology, floral characteristics, and collection details. Therefore, it is designated as the lectotype in this study.

***Impatiens commelinoides* Hand.‐Mazz., Symb. Sin. Pt**. **VII** **651 (1933)**

*Type*: China, Prov. Kianghsi, Steinige Stellen auf Kalk am Wuhwa‐scha bei Ningdu, c. 800 m, 21‐23 VII. 1921, wang‐te‐hui (holotype: WU!, WU0059595; isotype: E!, E00313651).

=*Impatiens fanjingshanica* Y.L.Chen syn. nov., Acta Phytotax. Sin. 37(1): 93 (1999).

*Type*: China, Prov. Guizhou, Fanjingshan, in declivo herboso humido, alt 0.1500 m, 1986‐06‐06, *Sino‐America Exped. 813* (holotype: PE; isotype: PE!, PE00039702, PE00039703; HUH, A00091412).

=*Impatiens tienchuanensis* Y. L. Chen syn. Nov., Acta Phytotax. Sin. 16(2): 49 (1978).

*Type*: China, Prov. Sichuan, Tianquan, alt 0.1200 m, 1938–10–18, Hu Wenguang, He Zhu 11,768 (holotype: PE!; paratype: PE!).

***Impatiens oxyanthera* Hook.f., Nouv. Arch. Mus. Hist. Nat. sér. 4, 10: 254 (1908)**

*Type*: China, Szechuan, Mont. Omei, E. H. Wilson, n. 4740 (holotype: K!, K000694033; isotype: A!, A00051211; P!, P00780675).

=*Impatiens piufanensis* Hook.f. syn. nov., Hooker's Icon. Pl. 29: t. 2869 (1908).

*Type*: China, Kweichau, Piu‐fa, J. P. Cavalerie 314 (holotype: E!, E00313603; isotype: K!, K000694588).

=*Impatiens forrestii* Hook.f. ex W.W.Sm. syn. nov., Notes Roy. Bot. Gard. Edinburgh 8: 339, descr. (1915).

Type: China, Yunnan, Tail Range, G.Forrest (lectotype: E!, E00313622; isolectotype: K!, K000694041; syntype: E!, E00848230).

## 4 Discussion

In the realm of classical taxonomic inquiry, the morphological characterization of species inherently involves a degree of subjectivity. Incorporating molecular‐level investigations is an effective approach to discerning the phylogenetic relationships among plant species. However, the study of inter‐species relationships is only meaningful when grounded in meticulous on‐site surveys of populations at the type localities and accurate specimen identification. Without these, further confusion may arise, especially for species with ambiguous taxonomic standing.

The structural attributes of the chloroplast genomes of the 10 *Impatiens* samples investigated in this study are largely congruent with findings from previous research on the genus. The IR boundaries exhibit relative conservatism, differing only subtly, and generally align with the phylogenetic relationships observed. As with most angiosperms, the majority of gene sequence variation occurs in the non‐coding regions. Specifically, within the SSC region, coding regions of genes such as *ndhF*, *ycf1*, and *ndhH* exhibit a relatively high degree of variability (Luo, Huang, et al. 2021). Comparative analyses with prior studies reveal that the identification of highly variable regions yields different results depending on the species under consideration. Nonetheless, it remains a consistent observation that the SSC region shows significantly more variation than other regions. Among these, *ycf1* demonstrates the highest Pi value, making it the most promising DNA barcode marker for the genus *Impatiens*.

Species that share similar morphological features typically exhibit closer phylogenetic relationships. The phylogenetic tree constructed from 32 chloroplast gene sequences reveals that the 10 species examined in this study are divided into three clades. Notably, within clade 1, the original description of *I. sunii* suggests its close relationship with *I. piufanensis*. Upon the publication of *I. piufanensis* var. *villosa*, the authors used chloroplast fragments (*atpB‐rbcL*) and ITS fragments from multiple populations to confirm the conspecificity of *I. piufanensis* var. *villosa* and *I. piufanensis*. *I. piufanensis* var. *villosa* is distinguished from the autonymic variety by its pubescent habit and the presence of a narrow‐winged protrusion on the vexillum. In the phylogenetic tree constructed using the chloroplast genome in this study, *I. piufanensis* shows a closer phylogenetic relationship with *I. oxyanthera*, while *I. sunii* is more closely related to *I. forrestii*. Both phylogenetic trees robustly support the clustering of these five species within the same clade (BS = 1.00). Extensive field surveys have shown that species in clade 1 are morphologically similar but exhibit varying degrees of variation across different populations. Plants manifest divergent morphologies depending on their habitats. For example, individuals growing near water bodies often develop swollen roots as an adaptation for fixation in flowing water. Interestingly, the protologue of some species within this group does not account for root swelling. Furthermore, morphological transitions in diagnostic features, as described in the original literature, are evident in closely related species. The species in this clade are primarily distributed in southwestern China, encompassing the Yunnan‐Guizhou Plateau. Their geographical distribution is continuous, lacking discernible geographical isolation. Consequently, these species are likely conspecific, and the scientific name *I. oxyanthera* should be retained. The distinctive and stable flower color of *I. sunii* and the pubescent trait of *I. piufanensis* var. *villosa* can reasonably be considered varietal characteristics within this taxon. However, wild populations of *I. sunii* were only discovered in Liupanshui City, Guizhou Province during this study. This locality is not its type locality (Daguan County, Yunnan Province), and no type specimens of this species were obtained for verification during the research process. Since taxonomic treatment relies on comprehensive evidence of locality traceability and comparative analysis of type specimens, to maintain nomenclatural stability, this study tentatively retains the species‐level taxonomic status of *I. sunii*, and its definitive taxonomic placement awaits further in‐depth research.

Clade 2 is also strongly supported by both phylogenetic trees (BS = 100). Geographically, *I. commelinoides* and *I. fanjingshanica* are widely distributed in southern China, particularly in the Guizhou Plateau and the high‐altitude regions of southeastern China. The geographical distribution in these areas is continuous, with no significant geographical isolation. In contrast, the type locality of *I. tienchuanensis* is located on the western margin of the Sichuan Basin, separated from the other two species by the basin itself, forming a distinct geographical barrier. The western margin of the basin is characterized by a subtropical monsoon climate, with altitudes ranging from approximately 1500–3000 m, conditions that align with the habitat of *I. commelinoides* and *I. fanjingshanica*. Notably, *I. fanjingshanica* is distributed along the southern margin of the Sichuan Basin, which exhibits a subtropical humid climate. The temperature gradient decreases from east to west, and the prolonged periods of hot, dry weather in the southeastern part of the basin make the region inhospitable for these *Impatiens* species. Thus, the isolation between the type localities of *I. tienchuanensis* and the other two species can be attributed to the unique geographical and climatic conditions of the Sichuan Basin. Field investigations have uncovered certain differences between *I. commelinoides* and *I. tienchuanensis*. However, these differences exhibit transitional forms in various populations of *I. fanjingshanica* that were not documented in the protologue. These subtle morphological discrepancies are likely the result of different life forms shaped by geographical location.

However, the validation and refinement of taxonomic treatments remain constrained by multiple practical limitations associated with sampling *Impatiens* species. Most *Impatiens* species are narrow‐range endemics, restricted to specific small‐scale habitats. Additionally, some wild populations are characterized by low abundance and sparse individuals; further, their succulent stems and thin, fragile leaves make specimen damage inevitable during sampling. The moist, shaded habitats they prefer are often fragmented due to human activities, while their occurrence in remote, high‐altitude areas poses challenges to field accessibility. In taxonomic research, two prevalent issues prevail: habitat destruction at type localities and the difficulty in accessing type specimens deposited in distant herbaria. These problems result in a lack of direct references for the taxonomic placement of collected samples. Moreover, genomic studies require fresh samples to be preserved at low temperatures—a requirement that is difficult to fulfill without adequate field equipment, leading to sample degradation and compromised DNA extraction quality. Future research may expand the number of populations included in sequencing to further elucidate the genetic diversity and divergence patterns of these species.

The newly described taxa, *I. xishuiensis* and its variety, are shown in the phylogenetic tree to have a closer phylogenetic affinity with *I. commelinoides*. Morphologically, they are more closely related to *I. oxyanthera*. The sub‐triangular labellum and the straight stripes on the sides make this species easily distinguishable. Moreover, these taxa exhibit a discordance between phylogeny and morphology. Morphologically, *I*. *xishuiensis* and *I. oxyanthera* share more distinct similarities, whereas phylogenetic analyses indicate that *I. xishuiensis* is more closely related to *I. commelinoides*. Given that the genus *Impatiens* is recognized as a group characterized by prominent mosaic evolution, we can reasonably hypothesize that the coexistence of plesiomorphies (ancestral traits) and apomorphies (derived traits) during the process of mosaic evolution has led to morphological convergence across different clades.

The Nayong population of *I. xishuiensis* exhibits a stable yellow color variation, which is also seen in *I. sunii*. Although no color polymorphism has been observed within the same population, its closely related species (with pink flowers) are widely distributed in Southwest China, showing continuity in their geographical distribution. Therefore, we propose that for some species of *I*. sect. *Impatiens*, flower color variation may be more susceptible to environmental factors or small‐scale genetic variations, and this trait may play a relatively limited role in accurately determining species taxonomic status.

## Author Contributions

**Qinqin Yong:** conceptualization (equal), data curation (equal), formal analysis (equal), investigation (equal), software (equal), visualization (equal), writing – original draft (equal), writing – review and editing (equal). **Xiao Wang:** investigation (equal), resources (equal), validation (equal), visualization (equal), writing – original draft (equal), writing – review and editing (equal). **Meijun Li:** project administration (equal), supervision (equal), validation (equal), writing – review and editing (equal). **Zhi Li:** conceptualization (equal), formal analysis (equal), supervision (equal), validation (equal), writing – review and editing (equal). **Xinxiang Bai:** conceptualization (lead), funding acquisition (equal), investigation (equal), project administration (equal), resources (equal), supervision (equal), validation (equal), writing – review and editing (equal). **Sheng Liang:** investigation (equal), resources (equal), supervision (equal). **Jinling Zhang:** funding acquisition (equal), supervision (equal), validation (equal), writing – review and editing (equal).

## Funding

This work was supported by Key Project of Guizhou Basic Research Program in 2026, Qiankehe Jichu ZD(2026)061, Special Fund for Innovation Capacity Construction of Guizhou Research Institution, Qiankehefuqi [2024]013, the 2024 Guizhou Science and Technology Innovation Talent Team Construction Project, Qiankeherencai CXTD[2025]053, National Natural Science Foundation of China, No. 32201282, Hainan Provincial Natural Science Foundation of China, No. 322QN249.

## Conflicts of Interest

The authors declare no conflicts of interest.

## Supporting information


**Table S1:** The sequence information of 32 loci used to construct the phylogenetic tree. (The species studied in this paper are highlighted in red font.)
**Table S2:** List of genes in the chloroplast genomes of the *Impatiens* species.
**Figure S1:** RSCU values of 20 amino acid and termination codons.
**Figure S2:** The ML tree constructed from the complete chloroplast genome.
**Figure S3:** The ML tree constructed from the CDS sequences.

**Table S3:** Codon usage patterns of the 10 chloroplast genomes.

**Table S4:** Best partitioning schemes and models for ML tree based on CDS.
**Table S5:** Best partitioning schemes and models for BI tree based on CDS.

## Data Availability

The chloroplast genomes of *Impatiens* assembled in this study have been deposited in GenBank of NCBI (https://www.ncbi.nlm.nih.gov). GenBank accession numbers: PQ877657—PQ877665. The sequence information used to construct the phylogenetic tree can be found in Table S1. The raw sequencing reads have been deposited in the NCBI Sequence Read Archive (SRA) database, with the corresponding SRA accession number: SAMN54211871—SAMN54211879.
