# Supplementary material for: Taxonomic Studies on 10 Species Within the Genus Impatiens Based on the Complete Chloroplast Genomes and Morphological Researches, Along With the Report of Newly Discovered Species
Source: Ecol Evol. 2026 Mar 18;16(3):e73279. doi: 10.1002/ece3.73279 (PMC13093384; doi:10.1002/ece3.73279)
Supplement: Supplementary file 1 — Table S1: The sequence information of 32 loci used to construct the phylogenetic tree. (The species studied in this paper are highlighted in red font.) Table S2: List of genes in the chloroplast genomes of the Impatiens species. Figure S1: RSCU values of 20 amino acid and termination codons. Figure S2: The ML tree constructed from the complete chloroplast genome. Figure S3: The ML tree constructed from the CDS sequences. [file ECE3-16-e73279-s003.docx]

**Table S1** The sequence information of 32 loci used to construct the phylogenetic tree.(The species studied in this paper are highlighted in red font.)

| Genus/Group name | Species name | NCBI accession number |
| --- | --- | --- |
| Hydrocera Blume | *Hydrocera triflora* | MG162585 |
| *I.* subg. *Clavicarpa* | *Impatiens guizhouensis* | MW411295 |
|  | *Impatiens tubulosa* | OR135492 |
| *I.* sect. *Unflorae* | *Impatiens mengtszeana* | MW727522 |
|  | *Impatiens hawkeri* | MN687854 |
|  | *Impatiens walleriana* | MW411299 |
| *I.* sect. *Racemosae* | *Impatiens cyanantha* | MW464332 |
|  | *Impatiens loulanensis* | MW411297 |
|  | *Impatiens uliginosa* | MN533984 |
| *I.* sect. *Impatiens* | *Impatiens lucorum* | OR135415 |
|  | *Impatiens liupanshuiensis* | PQ156318 |
|  | *Impatiens bijieensis* | PQ156316 |
|  | *Impatiens platychlaena* | OR135491 |
|  | *Impatiens lecomtei* | OR135432 |
|  | *Impatiens soulieana* | OR135419 |
|  | *Impatiens corchorifolia* | OR135477 |
|  | *Impatiens nubigena* | OR135425 |
|  | *Impatiens barbata* | OR135475 |
|  | *Impatiens imbecilla* | OR135437 |
|  | *Impatiens lateristachys* | OR135414 |
|  | *Impatiens baishaensis* | OR135410 |
|  | *Impatiens macrovexilla* | OK310515 |
|  | *Impatiens oxyanthera** | PQ877657 |
|  | *Impatiens piufanensis** | PQ877658 |
|  | *Impatiens forrestii** | PQ877659 |
|  | *Impatiens piufanensis* var. *villosa** | PQ877660 |
|  | *Impatiens sunii** | PQ877661 |
|  | *Impatiens fanjingshanica** | PQ877662 |
|  | *Impatiens commelinoides** | PQ877663 |
|  | *Impatiens tienchuanensis* | OR135417 |
|  | *I. xishuiensis-1** | PQ877664 |
|  | *I. xishuiensis-2** | PQ877665 |
| * represents newly sequenced data in this paper.  The 10 chloroplast genomes analyzed in this study are marked in red font. | | |

**Table S2.** List of genes in the chloroplast genomes of the *Impatiens* species

| Category of genes | Group of genes | Gene names |
| --- | --- | --- |
| Photosynthesis-related genes | Photosystem I | *psaA psaB psaC psaI psaJ* |
|  | Assembly and stability of Photosystem I | *ycf3* ycf4* |
|  | Photosystem II | *psbA psbB psbC psbD psbE psbF psbH psbI psbJ psbK psbL psbM psbN(pbf1) psbT psbZ* |
|  | Rubisco | *rbcL* |
|  | ATP synthase | *atpA atpB atpE atpF* atpH atpI* |
|  | Cytochrome b/f complex | *petA petB* petD* petG petL petN* |
|  | Cytochrome c synthesis | *ccsA* |
|  | NADPH dehydrogenase | *ndhA* ndhB*(2) ndhC ndhD ndhE ndhF ndhG ndhH ndhI ndhJ ndhK* |
| Transcription and translation-related genes | Transcription | *rpoA rpoB rpoC1* rpoC2* |
|  | Ribosomal proteins | *rpl2*(2) rpl14 rpl16* rpl20 rpl22 rpl23(2) rpl32 rpl33 rpl36 rps2 rps3 rps4 rps7 rps8 rps11 rps12*(2) rps14 rps15 rps16* rps18 rps19* |
| RNA genes | Ribosomal RNA | *rrn4.5(2) rrn5(2) rrn16(2) rrn23(2)* |
|  | Transfer RNA | *trnA-UGC*(2) trnC-GCA trnD-GUC trnE-UUC trnF-GAA trnfM-CAU trnG-GCC* trnH-GUG trnI-CAU(2) trnI-GAU*(2) trnK-UUU* trnL-CAA trnL-UAA* trnL-UAG trnM-CAU trnN-GUU(2) trnP-UGG trnQ-UUG trnR-ACG(2) trnR-UCU trnS-GCU trnS-GGA trnS-UGA trnT-GGU trnT-UGU trnV-GAC(2) trnV-UAC* trnW-CCA trnY-GUA* |
| Other genes | RNA processing | *matK* |
|  | Carbon metabolism | *cemA* |
|  | Fatty acid synthesis | *accD* |
|  | Proteolysis | *clpP1** |
|  | Translational initiation | *infA* |
| Genes of unknown function | Conserved reading frames | *ycf1 ycf2(2) ycf15(2*) |
| (2) indicates that m = number of repeat units = 2  *Gene contains intron | | |


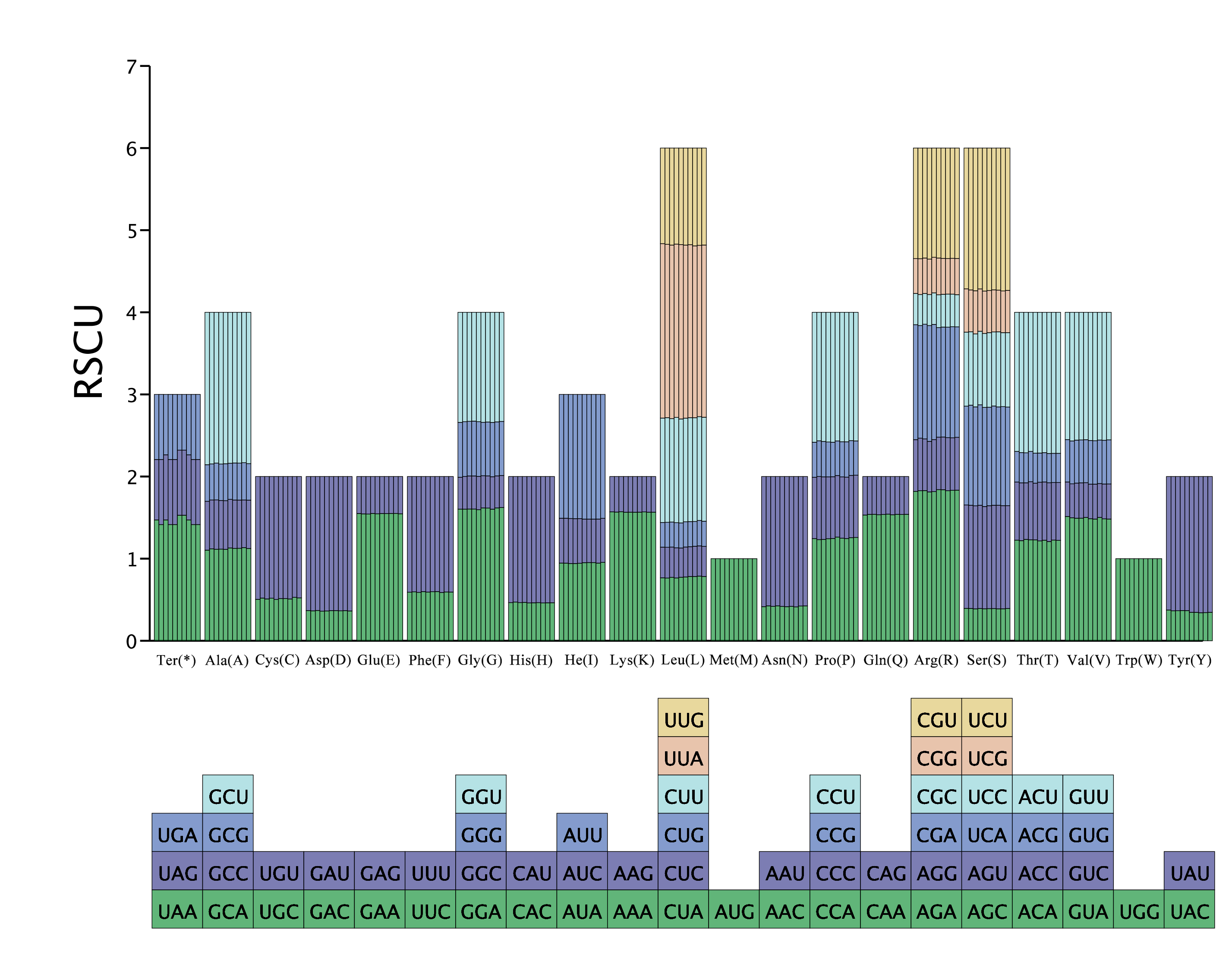


**Fig. S1** RSCU values of 20 amino acid and termination codons.


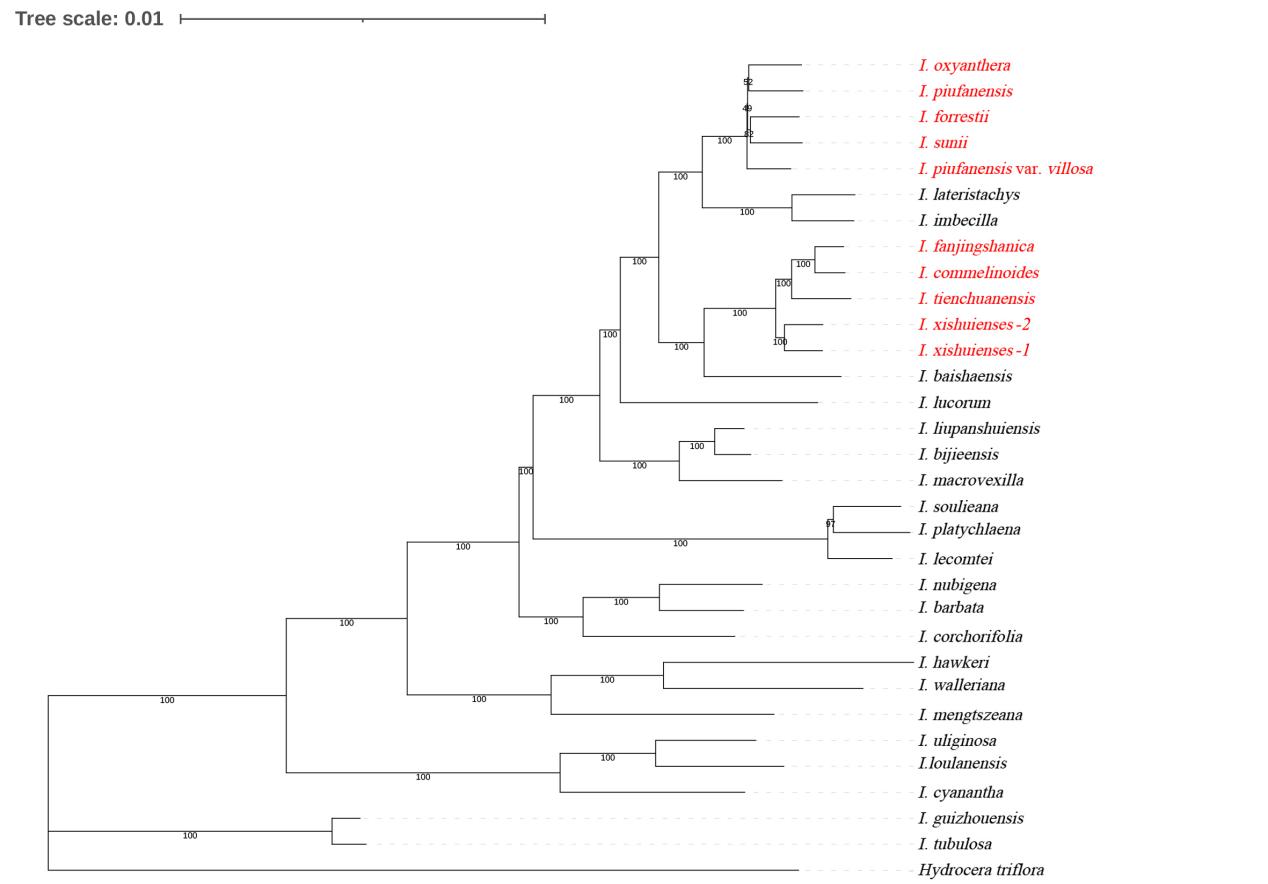


**Fig. S2** The ML tree constructed from the complete chloroplast genome.


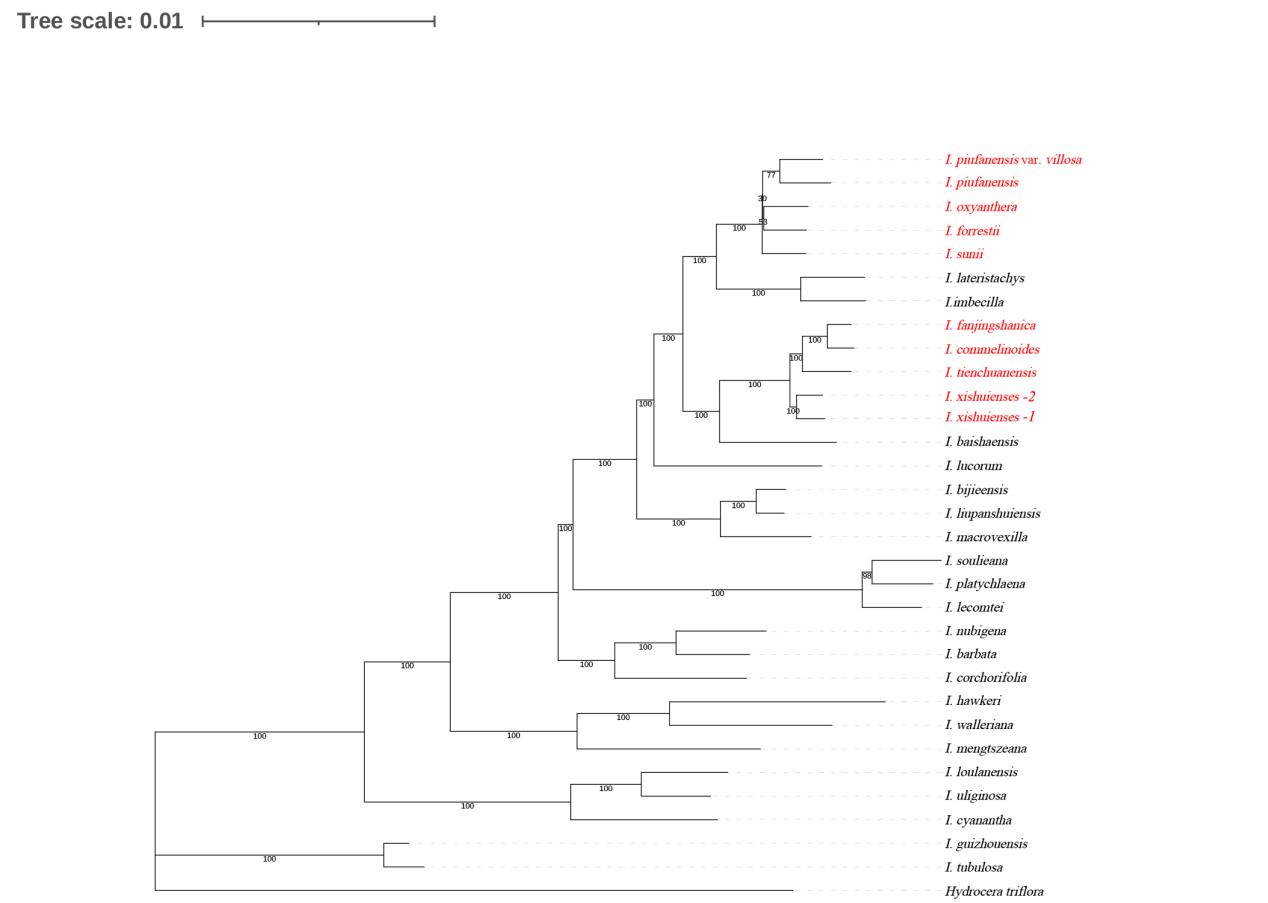


**Fig. S3** The ML tree constructed from the CDS sequences.
